# Neutrophils: Many Ways to Die

**DOI:** 10.3389/fimmu.2021.631821

**Published:** 2021-03-04

**Authors:** Erandi Pérez-Figueroa, Pablo Álvarez-Carrasco, Enrique Ortega, Carmen Maldonado-Bernal

**Affiliations:** ^1^Unidad de Investigación en Inmunología y Proteómica, Hospital Infantil de México Federico Gómez, Secretaría de Salud, Mexico City, Mexico; ^2^Departamento de Inmunología, Instituto de Investigaciones Biomédicas, Universidad Nacional Autónoma de México, Ciudad Universitaria, Mexico City, Mexico

**Keywords:** cell death, apoptosis, pyroptosis, necroptosis, necrosis, NETosis, autophagy, neutrophils

## Abstract

Neutrophils or polymorphonuclear leukocytes (PMN) are key participants in the innate immune response for their ability to execute different effector functions. These cells express a vast array of membrane receptors that allow them to recognize and eliminate infectious agents effectively and respond appropriately to microenvironmental stimuli that regulate neutrophil functions, such as activation, migration, generation of reactive oxygen species, formation of neutrophil extracellular traps, and mediator secretion, among others. Currently, it has been realized that activated neutrophils can accomplish their effector functions and simultaneously activate mechanisms of cell death in response to different intracellular or extracellular factors. Although several studies have revealed similarities between the mechanisms of cell death of neutrophils and other cell types, neutrophils have distinctive properties, such as a high production of reactive oxygen species (ROS) and nitrogen species (RNS), that are important for their effector function in infections and pathologies such as cancer, autoimmune diseases, and immunodeficiencies, influencing their cell death mechanisms. The present work offers a synthesis of the conditions and molecules implicated in the regulation and activation of the processes of neutrophil death: apoptosis, autophagy, pyroptosis, necroptosis, NETosis, and necrosis. This information allows to understand the duality encountered by PMNs upon activation. The effector functions are carried out to eliminate invading pathogens, but in several instances, these functions involve activation of signaling cascades that culminate in the death of the neutrophil. This process guarantees the correct elimination of pathogenic agents, damaged or senescent cells, and the timely resolution of the inflammation that is essential for the maintenance of homeostasis in the organism. In addition, they alert the organism when the immunological system is being deregulated, promoting the activation of other cells of the immune system, such as B and T lymphocytes, which produce cytokines that potentiate the microbicide functions.

## Introduction

Neutrophils, also denominated microphages or polymorphonuclear leukocytes (PMN), are granulocytes that are the most abundant leukocyte type in the peripheral human blood, constituting from 40 to 70% of the total white blood cells under normal conditions (1). Their half-life is short, from 8 to 20 h in circulation, although after migration into tissues, their life is considerably prolonged (1–4 days) (2). They comprise the first cells of the immune system to arrive at the infection site and are characterized by their potent antimicrobial and antifungal capacity, which is granted by their cellular components: antimicrobial peptides, neutrophil-specific proteolytic enzymes, as well as their production of ROS and neutrophil extracellular traps (NETs) (3–5).

Neutrophils contain an extraordinary array of receptors for the recognition of pathogens, including membrane receptors such as scavenger receptors (6, 7), mannose receptors (8), Dectin-1 (9), CD14 (10, 11), C1qR (12), receptors for IgG (FcγR) (13–16), C3b/C3biRs (CR1,CR3) (17), collectins (CD91-calreticulin complex) (18, 19), and Toll-like receptors (TLRs) (20); they also express intracellular receptors such as NOD-like receptors (NLRs) (21). Through these receptors, known as pattern recognition receptors (PRRs), neutrophils are activated to respond to infections or to damage signals. PMNs also contain enzymatic machinery for the production of free radicals that allows a much more effective elimination of pathogens (22). At the same time or after performing their effector functions, neutrophils initiate mechanisms of cell death such as autophagy, NETosis, or apoptosis. Cell death of neutrophils is an indispensable event required to maintain the number of neutrophils in infection or inflammation and in an organism in homeostasis (23–25).

The regulation of neutrophil death rate is an essential mechanism for maintaining homeostasis under physiological conditions. Their accelerated death, either idiopathic or acquired (e.g., drug-induced), leads to a decrease in the total count (neutropenia) increasing the probability of contracting bacterial or fungal infections and negatively affecting their resolution (26, 27). In contrast, delays in the death of neutrophils raises their count (neutrophilia) (28). Neutrophilia is a classic indicator of acute inflammation related to acute atherosclerotic events or trauma. For these reasons, the regulation of neutrophil survival is essential in the inflammatory process. The following review brings together recent findings related to the mechanisms that regulate the death of neutrophils, as well as the intrinsic and extrinsic signals that initiate these processes.

## Review

### Processes of Cell Death

#### Apoptosis

The cells of an organism are capable of dying through a mechanism of programmed cell death denominated apoptosis, characterized by changes in the cell structure, such as the acquisition of an elongated shape, retraction of the pseudopods, reduction of the cell volume (pyknosis), and chromatin condensation, with scarce or null structural modification of the cytoplasmic organelles. Other changes include DNA fragmentation with or without oligonucleosomal fragmentation, and usually activation of caspases (29–31). Apoptosis occurs with minimal damage to the surrounding tissue.

In neutrophils, this process is initiated by either of two distinct signaling pathways: the intrinsic or the extrinsic apoptotic pathway. In the intrinsic pathway, permeabilization of the mitochondrial membrane, due to the absence of antiapoptotic factors, leads to the release of cytochrome C to the cytoplasm. Once in the cytoplasm, the cytochrome C participates in the assembly of a multimeric complex denominated apoptosome and the subsequent activation of the effector caspase (caspase-3). On the other hand, the extrinsic pathway is activated in response to extracellular ligation of death receptors, resulting in the activation of caspase-3 by means of the activation of caspase-8 (28, 29, 32, 33).

In this first phase of the death process, the neutrophils are no longer functional since their chemotaxis, respiratory burst, and degranulation are impaired (34, 35), in addition to a decrease in the expression of surface receptors and adhesion molecules (36, 37) and a significant reduction in the release of chemokines (38).

It is also known that neutrophil apoptosis can be delayed or accelerated depending on specific cellular stimuli. According on the inflammation conditions, the neutrophil response to microorganisms can change significantly: while some pathogens initiate the spontaneous apoptotic process, others engage in such tight interactions that cause apoptosis followed by cell lysis (39, 40). The apoptotic death process, as opposed to the necrotic death process, maintains membrane integrity to limit the release of harmful neutrophil contents. The exposure of phosphatidylserine (PS) residues on the cell membrane allows for greater recognition by macrophages, thereby enhancing the clearance of the cells. It also increases the production of IL-10 and TGF-β in macrophages, cytokines associated with resolving the inflammatory response and promoting tissue repair (41, 42).

In contrast, the life span of neutrophils can be considerably extended under certain inflammatory conditions, such as in the presence of granulocyte-colony stimulating factor (G-CSF), granulocyte-monocyte colony-stimulating factor (GM-CSF), Interleukin 8 (IL-8), and C reactive protein, among other proinflammatory mediators (43–46).

Apoptosis is a non-inflammatory process of cell death activated in senescent neutrophils by cytokines and growth factors inducing the PI3-K/Akt, BCL-2/BCL-X_L_, and p53/p21 pathways, in a similar way as it occurs in other cells (32, 47, 48). However, this is not the only way to activate apoptosis in neutrophils. It is recognized that apoptosis pathways can be activated in neutrophils after phagocytosis and ROS production, or during inflammatory processes when molecules such as extracellular matrix proteins (49), lipopolysaccharide (LPS), complement fragments (44), and cytokines, among others, favor the activation of TNF receptors and FaS/CD95 through JNK and NF-kB (50, 51).

The high concentrations of reactive oxygen species produced by the NADPH oxidase are an important marker for triggering apoptosis or necrosis. In resting neutrophils, the NADPH is inactive and its components are distributed between the cytosol and cellular membranes. Upon neutrophil activation, the cytosolic components (p47phox, p67phox, p40phox) and the Rho family GTPase Rac-2 migrate toward the membranes of intracellular granules and the cell membrane (52) to participate in the assembly of the NADPH oxidase complex, which mediates the metabolic burst and ROS production. In the last years, it has been suggested that ROS produced by the NADPH oxidase participate as signaling molecules and it has been shown that the molecular pathways leading to apoptosis of neutrophils depend on ROS generation (53–55).

Kinases such as AKT and JNK regulate apoptosis-NETosis pathway in a ROS production-dependent manner. Activated AKT leads to NETosis, however its inhibition lead neutrophils to apoptosis (56) while JNLK is an important molecular sensor that initiates NETosis in response to increasing concentrations of LPS- and Gram-negative bacteria (57, 58). Recent studies show that apoptosis can occur along with NETosis, when neutrophils are stimulated with ultraviolet (UV) irradiation, calling it ApoNetosis. This type of death activates caspase 3 and is NOX-independent, though this interesting development requires further investigation (59).

#### Necroptosis

While apoptosis is one of the most studied forms of cell death in different cellular lineages, necroptosis is another type of programmed cell death that is not so well-known. This type of death is a regulated process, which depends on receptor interacting protein kinase-3 (RIPK3) and mixed lineage kinase domain-like protein (MLKL) (60–62).

While necroptosis is a process of programmed cell death, it does not involve the precise morphological changes characteristic of apoptosis and, importantly, it does not involve DNA fragmentation. Diverse forms of activation of neutrophils have been found to culminate in this process. Stimuli such as the activation of cell death receptors like TNFR1, ligation of TLRs, IFNGR, adhesion receptors such as CD11b, CD15, or CD18, the presence of monosodium urate (MSU) crystals, and the phagocytosis of *Staphylococcus aureus*, have been shown to initiate activation pathways that culminate in the activation of the RIPK3-MLKL complex (62–67). The signaling events upstream of the activation of the RIPK3-MLKL complex are different depending on the receptor involved. The activation pathway initiated through the TNFR1 is probably the best-characterized one (68). The activation of the receptor induces the formation of a complex with RIPK1, whose ubiquitination through diverse proteins, such as the cellular inhibitors of apoptosis [cIAP] and the linear ubiquitin chain assembly complex [LUBAC]) (69–71), activate the NF-κB survival factor, thus contributing to cell survival, proliferation, and production of proinflammatory cytokines; if ubiquitination of RIPK1 is inhibited, a different complex is formed that recruits caspase-8, inducing apoptosis (72); however, if the activity of caspase-8 is inhibited, then RIPK1 recruits RIPK3 in a complex called the necrosome. This complex can now recruit the caspase-8 in order to give rise to the necroptotic process (73–75).

In the case of activation of adhesion receptors or the presence of MSU crystals, the activation of necroptosis depends on the formation of ROS inside the cell, although the mechanism by which ROS induce the activation of the RIPK3-MLKL complex has not been not established. However, intracellular production of ROS is indispensable for necroptosis, as neutrophils of patients with chronic granulomatous disease are incapable of entering into necroptosis through the adhesion molecules (62, 76). In the case of MSU, there is evidence that relates necroptosis with the release of neutrophil extracellular traps (NETs); this stimulus is linked with the formation of ROS by inducing the assembly and activity of the NADPH oxidase complex. It was observed that the inhibition of RIPK3, as well as the action of other necroptosis inhibitors, diminished the formation of NETs induced by MSU crystals, thus confirming the relation of necroptosis with NETosis, at least after this stimulus (77).

#### Necrosis

Adverse environmental conditions, such as lack of oxygen or essential nutrients, high temperature, toxic compounds, and mechanical stress, can initiate necrosis. In contrast with apoptosis, there is a gain in cell volume (oncosis), rupture of the plasma membrane, and release of intracellular material to the surrounding milieu during necrosis (78, 79); similarly, there is swelling of cytoplasmic organelles (mitochondria, endoplasmic reticulum, and Golgi), moderate chromatin condensation, the absence of the activation of caspases, release of cytochrome c, and fragmentation of oligonucleosomal DNA. Morphologically, these have been defined as the spectrum of post-mortem changes of a tissue due to the progressive action of the enzymes released by the damaged structures (79). The morphology of the necrotic cells results from the denaturation of proteins and the autolytic or heterolytic enzymatic digestion (30, 31, 79).

The habitual participants in necrotic cell death, independently of the stimulus, are Ca^2+^ ions and ROS. During necrosis, the levels of cytosolic Ca^2+^ rise, leading to an overload of calcium in the mitochondria and the activation of proteases and phospholipases (80). ROS generate damage to lipids, proteins, and DNA; consequently, there is mitochondrial dysfunction, deregulation of the ionic equilibrium, and loss of membrane integrity. Destabilization of the membrane during necrosis is also mediated by other factors, such as acid sphingomyelinase (SMA), phospholipase A (PLA), and the calpains. In addition, necrotic cells release immunomodulatory factors that lead to their recognition and trapping by phagocytes, which can promote a subsequent immunological response.

Necrotic neutrophils, as well as other cells such as monocytes and dendritic cells, can activate NF-κB in neighboring cells (like fibroblasts or macrophages), stimulating the production of IL-6, IL-8, and TNF-α (81). The stimuli that induce this type of cell death of neutrophils are mainly pathogens that prompt a ligand–receptor response, giving rise to a high concentration of intracellular ions, as well as to the release of proinflammatory cytokines since the external stimulus. TNF-α secreted by activated neutrophils or other cells can activate neutrophil necrosis, although membrane proteins can regulate this with enzymatic activity, such as aminopeptidase N (CD13) (82, 83).

#### NETosis

In 2004, the discovery of neutrophils extracellular traps (NETs) established a mechanism of effector response specific of these cells (84). However, it was later demonstrated that all granulocytes possess this capacity after treatment with IL-5 and IFN-γ or with GM-CSF and C5a (85). The release of NETs is part of a programmed cell-death process denominated NETosis. In contrast with apoptosis or necrosis, the nuclear membrane and the cellular membrane disintegrate; however, the cytoplasm contents remain integrated for a moment, allowing the interaction of the antimicrobial granule proteins with the DNA (86, 87). On releasing its genetic material, the extracellular chromatin functions for immobilizing the bacteria and preventing its dispersion through the host tissues. Simultaneously, these traps are capable of degrading virulent bacterial products like IcsA, a key virulence factor of the human pathogen *Shigella flexneri* (88) or IpaB (invasive plasmid antigen B) (89, 90). These bacterial products and their subproducts can induce inflammation and diverse chemotactic signals that result in the recruitment of monocytes and other cells to the infection site (84, 86, 91).

The process of NETosis can be induced by an extensive number of stimuli that include pathogenic microorganisms, activated platelets (92), pathogen's structures, formylated peptides, and cytokines, among others. Signals characteristic of apoptosis, such as the condensation of genetic material, the presence of phosphatidylserine (PS) in the membrane, and activation of caspases, are not observed during NETosis (93).

This process is regulated, among others, by peptidyl arginine deaminase 4 (PAD4), an enzyme that contains a putative sequence of nuclear localization, but that resides principally in cytosolic structures in resting neutrophils, although it can also enter into the nucleus and citrullinate the histones and diverse transcription factors. Therefore, it can participate in the epigenetic regulation of gene expression and cellular differentiation (94, 95). PAD4 is associated with the cytosolic subunits p47^phox^ (also known as neutrophil cytosolic factor 1, NCF1) and p67^phox^ (NCF2) that form part of the NADPH oxidase complex involved in the respiratory burst. Activation of the cell leading to the elevation of intracellular Ca^2+^ to physiological levels do not result in activation of PAD4 enzymatic activity. However, high levels of intracellular calcium (higher than those of physiological neutrophil activation, such as those observed by disruption of the cell membrane) lead to activation of PAD4 and rapid citrullination of p47phox/NCF1 and p67phox/NCF2, as well as their dissociation from PAD4. Citrullination of NCF1 and NCF2 prevents the assembly of NADPH oxidase complex (95). Originally, it was reported that the presence of NADPH oxidase and myeloperoxidase (MPO) was necessary for the correct formation of NETs; until recently, all the activators capable of inducing NETs formation were known to require in some manner the presence of ROS (96). However, this has been a controversial theme in recent years due to the description of novel NETosis activation pathways that appear to be independent of NADPH oxidase (81, 97, 98).

PAD4 citrullinates chromatin, specifically histones, catalyzing the deimination of the arginine at the amino terminal end of the H3 subunit. This reduces the attraction of heterochromatin protein HP1B to H3-adjacent lysine 9, resulting in the formation of dispersed chromatin. In addition to this, neutrophil elastase (NE) and MPO complement the decondensation of chromatin. Both NE and MPO are stored in azurophilic granules that are released once ROS are formed in the cell. NE is a PMN-specific serine protease that degrades virulence factors and is bactericidal. However, during the process of NETosis, it enters the nucleus and cleaves the histones, promoting greater chromatin decompactation (99). Later during the NETosis process, when the NE have already acted, MPO binds to the chromatin prompting even a greater decompactation. The synergic process of these enzymes for the formation of dispersed chromatin reaches the point at which the nuclear and cellular membranes break and NETs are released (100). Interestingly, the most abundant component of the NETs are histones and these are one of the most potent antimicrobial factors (96, 101).

Activated neutrophils produce large amounts of superoxide through the NADPH oxidase. Superoxide is then converted into hydrogen peroxide (H_2_O_2_) leading to the formation of a variety of toxic oxygen derivatives. MPO catalyzes the oxidation of halides by means of hydrogen peroxide. Both, the NADPH oxidase and MPO, have been implicated in regulating NETs formation. It is now known that the ROS generated through the NADPH oxidase prevent activation of caspases (102) and that NADPH oxidase is necessary for the formation of NETs through many (though probably not all) stimuli.

Participation of the Raf-MEK-ERK pathway in the formation of NETs has been identified by the phosphorylation of ERK1, ERK2, and c-Raf. Since the inhibition of the NADPH oxidase does not block the phosphorylation of these proteins induced by PMA, this suggests that activation of the Raf-MEK-ERK pathway is found upstream of NADPH oxidase activation (103, 104).

NETs formation depends directly on the intensity of the stimulation of the cell and on the time of exposure to the stimulus. Currently, NETosis continues to be considered a process of cell death, although some studies have demonstrated a process known as vital NET formation, in which neutrophils release mitochondrial DNA, which does not compromise their viability (105, 106).

#### Pyroptosis

This pathway of cell death is dependent on caspase-1. This caspase is not involved in apoptotic cell death and its function is to process the precursors of the IL-1β and IL-18 inflammatory cytokines through the multiprotein complex called inflammasome (107, 108). This type of cell death is observed, e.g., in cells infected with *Salmonella*, in which the activation of caspase-1 is produced by effector substances released into the cytoplasm of host0 cells through the *Salmonella* Pathogenicity Island 1 Type III Secretion Systems (SP1 T3SS).

The assembly and function of an inflammasome are essential for the process of pyroptosis; these oligomeric complexes serve as a molecular platform for the key step of the process, that is, the cleavage of pro-CASP-1 zymogen into active caspase-1. Canonical inflammasomes are composed of cytoplasmic pattern recognition receptors (PRR), which include NLRs, absent in melanoma 2 (AIM-2)-like receptors (ALRs), adapter protein apoptosis-associated speck-like protein containing a CARD (ASC), and pro-caspase-1 (109, 110). Numerous canonical inflammasomes have been recognized in pyroptosis including NLRP1, NLRP3, AIM2, NLRC4, and Pyrin inflammasomes (111–116).

Activation of caspase-1 results in the formation of pores in the plasma membrane and the cell becomes permeable to small-molecular-weight colorants such as 7-aminoactinomycin (7-AAD), ethidium bromide (EtBr), and propidium iodine (PI). In contrast, the cell membrane in an apoptotic cell remains intact and the cells fragment into apoptotic bodies that are not stained with 7-AAD nor with PI (108). Activation of caspase-1 leads to DNA fragmentation and to cell lysis by separate pathways. DNA fragmentation is carried out by an unidentified caspase 1-activated nuclease that does not involve the degradation of ICAD (inhibitor caspase-activated deoxyribonuclease) (117, 118) that, together with rearrangements of the actin cytoskeleton, are events required for the formation of membrane pores of between 1.1 and 2.4 nm in diameter (119). In addition, caspase-1 cleaves the precursors to produce biologically active IL-1B and IL-18, which come out easily through the pores. Dissipation of the cellular ionic gradients leads to a flow of water with cellular tumefaction and osmotic lysis (120), releasing the intracellular (proinflammatory) contents. Even so, according to some researchers, pyroptosis comprises nothing more than a caspase-1-dependent necrotic-type death.

Pyroptosis has been mainly described in macrophages and dendritic cells. However, there is evidence of caspase-1 activity in other cell types. Many pathogens can survive and replicate within the macrophages; however, very few pathogens can do this in neutrophils. This is probably due to intrinsic differences between the two cell types. Macrophages have a longer life but reduced microbicidal activity as compared to neutrophils, which converts them into a more infection-prone cellular objective. Neutrophils, on the other hand, are highly microbicide and short-lived, which makes them poor targets for intracellular pathogens.

During acute Salmonella infection, neutrophils can activate the NLRC4 inflammasome to produce IL-1β, without undergoing pyroptosis, and thus they can continue their inflammasome-independent antimicrobial effector functions while maintaining IL-1β secretion (121, 122). This can suggest that neutrophils do not undergo pyroptosis in response to proteins of some intracellular pathogens (such as flagellin or the proteins making up T3SS) that activate assembly of NLRC4 inflammasome. However, it has been determined that neutrophils can undergo pyroptosis, activating a non-canonical infammasome pathway through P2X7 receptors in absence of NOX2 in pseudomonal infections (105, 123, 124).

#### Autophagy

The term autophagy derives from the Greek to eat (*phagy*) oneself (*auto*), that is, self-digestion. This is a highly conserved process in evolution that takes place in all eukaryotic cells, from yeasts to mammals. It occurs in response to different forms of stress, such as lack of nutrients, absence of growth factors, infectious processes, or hypoxia. Therefore, it is possible that the main function of this process is to provide necessary nutrients to maintain the vital functions of the cell in situations of energy or nutrient deprivation. Additionally, some studies point out that the process denominated “selective autophagy” is also utilized to remove “dangerous” material in the cytoplasm, such as damaged mitochondria or protein aggregates (125). To date, three distinct forms of autophagy have been identified, macroautophagy, microautophagy, and selectiveautophagy (126).

Autophagy is characterized by the formation of autophagic intracellular vesicles and the degradation of cellular contents, including essential organelles such as mitochondria, inside the vesicles. Many of the PRRs, including TLRs and NLRs, induce autophagy in macrophages (127). Similarly, neutrophils undergo autophagy in response to TLR ligands. Apparently, autophagy in neutrophils can be induced dependently or independently of phagocytosis (128, 129).

Autophagy in the neutrophil is a mechanism of survival. Pliyev and Menshikov (130) demonstrated that inhibitors of autophagy, such as 3-methyladenine (MA) and chloroquine (CQ), notably accelerate the spontaneous apoptosis of neutrophils as evidenced by exposure of phosphatidylserine, DNA fragmentation, and the activation of caspase-3. It has also been observed that human neutrophils can be induced to autophagy by simultaneous stimulation by sialic acid binding immunoglobulin-like lectin-9 (Siglec-9) ligation and certain cytokines with survival-promoting activity in neutrophils (131). Some studies have demonstrated that adhesion molecules induce death in neutrophils independently of caspases. The process is characterized by great cytoplasmic vacuolization and the fusion of organelles, thus, it has been associated with autophagy (132) Neutrophils with morphological signs of autophagy have been observed in septic shock, cystic fibrosis, rheumatoid arthritis, and various skin diseases, suggesting that the induction of autophagy in polymorphonuclear leukocytes comprises a general phenomenon in their response to inflammation (133). It has also been demonstrated that the inhibition of autophagy diminishes NETosis, avoiding chromatin decondensation and resulting in a cell death characterized by signs of apoptosis (134, 135).

Autophagy in neutrophils is activated by phagocytosis of pathogens or activation of pattern recognition receptors (PRRs) that induce non-canonical selective autophagy during bactericidal activity by pathogen-derived toxins and molecules that damaged organelles such as mitochondria or peroxisomes resulting in specific fusion of lysosomes with these organelles (136–139).

The fact that LPS or phagocytosis of opsonized particles can induce autophagy in neutrophils independently of the formation of NETs, suggests that it is an additional effector mechanism that is independent of other responses to pathogens (140); however, the molecular mechanisms responsible for this remain unclear and in dispute. In this manner, bacteria that could escape from the phagolysosome may become exposed to the degradative proteases present in autophagosomes (132).

#### Phagocytosis

Phagocytosis is an important physiological mechanism that allows the elimination of excess cells or damaged cells and, in cases of infection by pathogens, their elimination. Because many of the eat-me signals are displayed on necrotic cells or cells in the process of apoptosis, it was thought that phagocytes only feed on dead cells or cells condemned to die (141). However, there is now evidence that phagocytosis of viable cells can cause their death, a process that has been termed primary phagocytosis or phagoptosis (142, 143).

The process of phagocytosis of apoptotic neutrophils is normally initiated with exposure of the phospholipid, phosphatidylserine (PS) (the “eat-me” signal). In healthy cells, PS is found nearly exclusively in the internal leaflet of the plasma membrane but, in apoptotic cells, it becomes exposed on the outer side through the action of a phospholipid scramblase. Exposure of PS can take place because of the following diverse processes:

The increase of intracellular Ca^2+^ ions that stimulates the scramblase and inhibits the translocase of phospholipids (flippase).
Depletion of ATP, which inhibits the translocase.
Oxidative stress, which stimulates the scramblase and inhibits the translocase.
The fusion of intracellular vesicles with the plasma membrane.
Necrosis due to the rupture of the plasma membrane, an increase of intracellular calcium or depletion of ATP (144, 145).

Activated macrophages can induce the exposure of PS on viable neutrophils (independently of apoptosis). PS exposure can then promote their phagocytosis, which is facilitated by the PS-binding proteins MFG-E8 (Milk fat globule-EGF factor 8 protein) and Annexin 1 (146).

The plasminogen activator inhibitor 1 (PAI-1) acts as a “don't-eat-me” signal in neutrophils as phagocytosis of viable neutrophils of PAI-1 knockout mice by macrophages was increased when compared to that of neutrophils from wild type mice. On the other hand, when PAI-1 or its receptor (CD47) are blocked, calreticulin (CTR) acts as the main “eat-me” signal and the lipoprotein receptor-related protein (LRP) (147) mediates phagocytosis.

Changes in the microenvironment of the neutrophil after having initiated a phagocytic process can lead to cell death. Neutrophils are exceptionally efficient phagocytes and are capable of gobbling up a prey opsonized with IgG in <20 s, in comparison with macrophages, which need up to several minutes to ingest similar amounts of opsonized particles (148). In neutrophils, the recruitment of NADPH oxidase during phagosome maturation is significantly higher than in macrophages due to the fusion of cytoplasmic granules with the phagosome (149, 150). These, and probably other mechanisms, result in the neutrophil phagosome to retain a pH close to neutral for longer periods of time, in comparison with the maturation of the phagolysosome in macrophages, in which acidification can reach pH values of 4.5–5 (151, 152).

It was reported that after phagocytosis of heat-killed *Escherichia coli*, the intracellular pH is crucial to determine the fate of the neutrophil and that the pH depends on the pathogen: phagocyte ratio; mild acidification of pH leads to cell apoptosis, but conditions of high bacterial concentrations that induce a larger decrease in pH lead to necrosis (153). In the case of virulent *Staphylococcus aureus* strains, it was shown that its phenol-soluble modulin (PSM) alpha proteins cause necrosis of neutrophils after phagocytosis, increasing the survival rate of the bacteria (154, 155). It is known that TNFα, influenza virus, as well as the virulence factors of *E. coli, Mycobacterium tuberculosis*, and other microorganisms, promote the death of neutrophils (156–158). In the majority of cases, this occurs after certain virulent factors are able to escape from the phagosome, activating cell death pathways such as necrosis or pyroptosis. As mentioned earlier, pyroptosis is mediated by the inflammasome, which is assembled after the phagocytized bacteria escape from the phagosomes and the bacterial products are detected by PRRs in the cytosol (159, 160).

## Intracellular Mediators of the Cell Death of Neutrophils

### Free Radicals

In a normal process and under the appropriate stimuli, neutrophils are capable of releasing cytotoxic mediators, such as ROS and RNS, generating damage to pathogens but also to the host's tissues. After the elimination of the proinflammatory stimuli, repaiment of the damaged tissue is necessary to return the tissue to a homeostasis state. At this point, anti-inflammatory signals start to be released contributing to the resolution of inflammation, and neutrophils in the tissue should enter apoptosis and be ingested by macrophages in order to clean the inflamed area. Apoptosis of neutrophils is regulated by intracellular mediators and extracellular signals; among the intracellular mediators are the ROS, mainly produced by the NADPH oxidase of the activated neutrophils (161, 162), although some reports show that ROS can be generated by mechanisms that are independent of the NADPH oxidase. Thus, it was reported that the production of superoxide and hydrogen peroxide can be mediated by low-conductance, calcium-activated potassium channels known as SK (small conductance) channels (163, 164). Another way of generating ROS in the neutrophil is through the accumulation of electrons of the respiratory chain, linked to a low activity of complex IV due to the low levels of cytochrome c (16, 165–167). Decreasing intracellular ROS levels by reduced glutathione (GSH), as well as by catalase, inhibits the death of neutrophils (39, 168, 169).

ROS have the capacity to damage DNA generating cell death through direct or indirect activation of p53 (170). ROS can also induce activation of the inflammasome, formed by NLRs, an adaptor molecule, and caspase-1 (171, 172). However, ROS can directly alter the activity of the intracellular signaling pathways implicated in the death and survival of neutrophils, such as NF-κB and MAPK (mitogen-activated protein kinases) (173, 174). In fibroblasts, ROS cause oxidation and inhibition of JNK-inactivating phosphatases, promoting JNK activation, release of cytochrome c, and the subsequent activation of caspase-3 (175, 176). Similarly, it has been demonstrated that the accumulation of ROS results in the grouping of death receptors in lipid rafts and the activation of caspase-8, independently of the Fas ligand (29). The generation of intracellular (but not phagosomal) ROS, caused by neutrophil activation, triggers the process of apoptosis, while the production of intraphagosomal ROS during phagocytosis does not have this effect. This suggests that the location of the production of ROS is fundamental for inducing the death of neutrophils (177). Among the molecules involved in the regulation of ROS production after cell activation, is Bruton's tyrosine kinase (Btk). The absence of this kinase induces a greater production of ROS by hyper phosphorylation and activation of phosphatidylinositol-3-OH-kinase (PI3K) and protein tyrosine-kinases (TKPs) (178). In addition, the levels of free radicals can be elevated by nitric oxide synthase (NOS) through the production of nitric oxide (NO) (177, 179). In recent years, it has been demonstrated that NO has also the capacity to induce apoptosis in neutrophils (180). It has been shown that the derivative of oxatriazol-5-amine, PGE 3162, and SIN-1 increase the rate of apoptosis in human neutrophils, correlating with data of the greater DNA fragmentation and cell death in neutrophils treated with exogenous NO (181, 182). Interestingly, high levels of ROS or RNS in neutrophils inhibits the activity of the caspases, suggesting the existence of an alternative, caspase-independent cell-death pathway (177, 179, 183, 184).

### Caspases and Calpains Involved in Apoptosis

Most stimuli that lead to apoptosis converge in the mitochondria inducing the permeabilization of their external membrane. With permeabilization, a series of proteins are released that activate the caspases (185), which carry out the majority of the proteolytic events of apoptosis and are considered as the ultimate responsible for cell death. Caspases are localized in the form of procaspases in the cytoplasm and the intermembrane space of the mitochondria. Procaspases are activated by cleavage and act as executors cleaving cellular survival molecules triggering processes that induce cell death. Caspases are regulated at the post-translational level and possess a classic structure consisting of a prodomain (N-terminal), a small subunit (p10) in the C-terminal, and a large subunit (p20) that contains the active center with cysteine within a QACXG-conserved motif (186, 187).

The initiator caspases possess prodomains larger than effector caspases. The latter contain caspase recruitment domains (CARD), as in the case of caspase-2 or caspase-9, or cell death effector domains (DED), as in the case of caspase-8 and caspase-10, which permits them to interact with other molecules that regulate their activation. The stimulus for cell death induces the grouping of the initiator caspases (Scaffold-Mediated Activation), allowing them to transactivate by cross-cleavage and, thus, proceed to activate the effector caspases. In all the studied cases, the mature enzyme is a heterotetramer that contains two p20/p10 heterodimers and two active centers (186). In neutrophils, caspases 3 and 10 play an important role in the induction of cell death (188). In *Helicobacter pylori*-infected neutrophils, the activity of caspase-1 is increased, promoting its association into inflammasomes to participate in the triggering of pyroptosis (189). In neutrophils, the activity of caspase-9 also increases drastically during apoptosis (167).

The calpains are calcium-dependent cysteine proteases that can be divided into two subfamilies according to the cation concentrations necessary for their activation: micromolar for the μ-calpains and millimolar for the m-calpains. They play an important role in cell proliferation, progression of the cell cycle, cell migration, and programmed cell death (190). Calpains are present in the cytosol as inactive enzymes that are activated in response to increases in the cellular concentration of Ca^2+^ ions. Once activated, they can modify transcription factors, cytoskeletal proteins, kinases, and proapoptotic proteins, such as Bid and Bax, into fragments incapable of interacting with antiapoptotic proteins, inducing the release of cytochrome c and activation of caspase-3 leading to apoptosis (191, 192). In human neutrophils, calpains do not only play a role in apoptosis, but calpains are also involved in the adhesion of TNF-α-stimulated cells, and play a role in migration regulated by the cytosolic Ca^+2^ concentration (193, 194).

#### Bcl2 and IAP (Apoptosis-Regulatory Proteins)

The process of apoptosis in human cells is regulated by a family of diverse pro-apoptotic and anti-apoptotic proteins, with protein Bcl-2 as the prototype protein. Members of this family are grouped into three subfamilies as follows: the antiapoptotic proteins (Bcl-2, Bcl-Xl, Mcl-1, and others), the “multidomain”-type proapoptotic proteins (Bax and Bak), and the “BH3-only”-type proapoptotic proteins (Bid, Bim, Bad, among others). The balance among these three groups determines susceptibility to cell death or survival as shown by the increased resistance to apoptosis of certain tumors related to the overexpression of the antiapoptotic proteins.

Another family of proteins with the ability to inhibit apoptosis (denominated IAPs) regulate the cytochrome c-caspase pathway. In humans, the following three members have been characterized: XIAP: c-IAP 1, and c-IAP 2, which bind to caspase-9 preventing its activation. In neutrophils, caspase-9 increases its activity during apoptosis, even though the levels of cytochrome c in these cells are very low (165–167, 195). Data obtained by Murphy et al. (196) suggest that neutrophils possess a lower threshold of cytochrome c for the assembly of functional apoptosomes and their low content of cytochrome c can be compensated for by the elevated expression of apoptotic protease-activating factor 1 (Apaf-1). These further suggest that neutrophils retain a low expression of cytochrome c for the assembly of functional apoptosomes rather than for oxidative phosphorylation.

Neutrophils contain few mitochondria and these are found as tubular networks, and are grouped and depolarized under apoptotic conditions. Despite the relatively low levels of cytochrome c in these cells, the mitochondrial death pathway is functional (167, 197). In neutrophils, the expression of antiapoptotic molecules, such as Mcl-1 and Bcl-xl, is preferential in comparison with those of the proapoptotic protein family (198).

One of the mechanisms that regulate the transcription of the antiapoptotic proteins in neutrophils is stimulation with GM-CSF and TNF-α, which prevents the time-dependent nuclear localization of Mcl-1, and an increase in the transcription and translocation of Bcl-Xl, via stimulation of the NF-κB pathway. However, these cytokines also participate in the increase and maintenance of RNAm levels of BH3-only proapoptotic proteins. GM-CSF is a survival factor for neutrophils that promotes the transcription of Bim and the expression of BimeL (199). During neutrophil cell death by apoptosis, Mcl-1 levels diminish gradually, leading to the release of Bax and to its later translocation to the mitochondrial membrane (200). The amount of Mcl-1 can be regulated at the transcriptional level through NF-κB and PI3K (201). Another mechanism of regulation of the neutrophil mitochondrial integrity is the flow of potassium (K). A high concentration of extracellular K^+^ promotes the survival of neutrophils through the prevention of mitochondrial dysfunction and the release of proapoptotic factors.

## Modulation of Cell Death by Extracellular Stimuli

Trans endothelial migration (exiting the blood vessel to reach the inflammation site) is one of the situations that promote neutrophil survival. At the inflammation site, neutrophils encounter various cytokines and chemokines that promote their activation and survival. Inflammatory mediators delay the spontaneous apoptosis of neutrophils, contributing to the persistence of inflammation. The mechanisms responsible for this delay, and for their increased resistance to apoptosis induced by anti-Fas antibodies, are related to the inhibition of caspase activity (202). Interferons type I and II delay neutrophil death through the activation of STAT3 and the positive regulation of the expression of the inhibitor of apoptosis protein 2 (cIAP) (203).

TLRs ligands, such as LPS, R-848, and CpG, delay spontaneous neutrophil apoptosis through the activation of Toll-like receptors (TLRs). Delay of neutrophil cell death is associated with greater phosphorylation of Akt and an increase in the levels of the antiapoptotic proteins Mcl-1 and A1 (204). Stimulation with LPS also regulates the decreased expression of many other factors related to apoptosis, suggesting that gene regulation can play an essential role in the modulation of neutrophil death (205). The lifespan of neutrophils can be also prolonged by G-CSF and GM-CSF. GM-CSF can up regulate via PI3K/Akt the expression of antiapoptotic molecules, and down regulate the expression of proapoptotic molecules (206–208).

G-CSF can act by blocking Bid/Bax (195) and inhibiting the activation of caspase-3 (209). In a similar fashion, Bruno et al. (210) demonstrated in *in-vitro* experiments that the activation of the leptin receptors expressed on the membrane of neutrophils delayed their apoptosis. In addition, it was reported that high concentration of extracellular K+ ions prevents K+ efflux and delays neutrophil's apoptosis, suggesting that potassium released from damaged cells can function as a survival signal (211). Neutrophil death can also be delayed by activated endothelial cells through the release of GM-CSF and/or by direct interaction (212, 213). Similarly, neutrophil death is delayed by the interaction with natural killer (NK) cells, through the secretion of IFN-γ and GM-CSF by the latter cells (214). These studies showed that neutrophil death can be delayed by their interaction with other cells, not only by the action of secreted cytokines, but also as a consequence of integrin signaling induced by direct cell-cell interaction (215).

Another molecule with an antiapoptotic function on neutrophils is the plasminogen activator inhibitor-1 (PAI-1), which is present in severe inflammatory states and is associated with neutrophil activation. PAI-1 activates antiapoptotic-signaling pathways, including the positive regulation of PKB/Akt, Mcl-1, and Bcl-x (L) (216). On the other hand, TNF-α plays a dual role in neutrophils: it can induce either their survival or death, and the effect is dependent on the intracellular, as well as on the extracellular environment (201). Survival tends to be favored at low doses of TNF-α, while proapoptotic effects are observed at high concentrations (217–219).

Induction of cell death induced by TNF-α is mediated through its receptors by the activation of the JNK/MAPK pathways, release of ROS, and activation of caspase-8. However, cell death in the neutrophil can be caspase-independent (220). The TNF-α receptors can also promote survival in human neutrophils, inhibiting spontaneous death by inducing an increase in PKCδ and PI3K activity (221) and promoting the expression of the antiapoptotic molecules Al and Bcl-XL through an NF-κB-dependent pathway (222, 223).

## Cell Death Receptors

Apoptosis can be initiated by extracellular signals received through distinct membrane receptors known as death receptors or extrinsic pathway receptors. Two families of receptors have been identified as death receptors: the Fas protein (also called CD95 or APO-1) and receptors for TNF-α.

The transmembrane Fas protein has an intracellular death domain that, when Fas is aggregated, interacts with the adaptor protein FADD (Fas-associated protein with death domain), which participates in cell death activating caspases 8 and 10 (224). Additionally, the intracellular domain of Fas can activate another factor called DAP6 (death domain-associated protein 6, or Daxx). DAP6 activates a protein kinase pathway leading to the opposite effect, stimulating entering into the cell cycle and mitosis (225).

The Fas pathway remains inactive until the extracellular domain binds to Fas ligand (Fas L), a protein expressed on the membrane of a neighbor cell that initiates the apoptosis pathway in the cells expressing Fas. Since this apoptotic pathway does not require *de novo* protein synthesis, it acts very fast inducing apoptosis.

The membrane receptor 1 for TNF (TNFR1) is a member of the TNF receptors superfamily that has in its intracellular portion a death domain, thus being capable of inducing cell death. Upon activation, its intracellular domain interacts with proteins such as TRADD (TNF receptor associated death domain) and RAIDD (226) (receptor associated interleukin death domain), which activate the caspases that can initiate apoptosis (227). However, it can also associate with a different factor denominated TRAF (TNF receptor associated factor), which activates protein-kinases and stimulates cell proliferation instead of apoptosis (228).

Neutrophils constitutively express high levels of Fas and Fas L on their membrane; thus, they are highly susceptible to cell death. The activation of Fas leads to an increase in the activation of caspases 8 and 3, and increases mitochondrial permeability (229). Fas signaling also increases mitochondrial permeability through Bid, Bak, and Bax, accelerating the apoptosis of neutrophils, but they are not necessary for apoptosis to be produced. Apoptosis triggered by Fas can be inhibited by Mcl-1 and Bcl-2 (230). The use of mitochondrial stabilizers significantly suppresses Fas-mediated cell death, suggesting that mitochondrial alteration is an essential step for neutrophil apoptosis induced by Fas (229). Nonetheless, despite the large body of data showing that activation of the Fas/FasL pathway accelerates the death of neutrophils, there are studies conducted in Fas (lpr)- or FasL (gld)-deficient mice that show that the deficiency of either of these molecules does not alter the rate of neutrophil death. This suggests that FasL/Fas-mediated apoptosis is not essential in determining the lifespan of neutrophils during an acute inflammatory response (231, 232). In agreement with this, the blockage of Fas/FasL with specific antagonists did not alter the rate of spontaneous death of neutrophils (233).

Neutrophils also express other receptors, such as TRAIL-R2 and R3 and its ligand, the TNF-related apoptosis inducing ligand (TRAIL) (234, 235). It has been demonstrated that the exposure of neutrophils to exogenous TRAIL accelerates the process of cell death and it has been suggested that this ligand suppresses cytokines with antiapoptotic effects (236–239).

**Figure 1:**
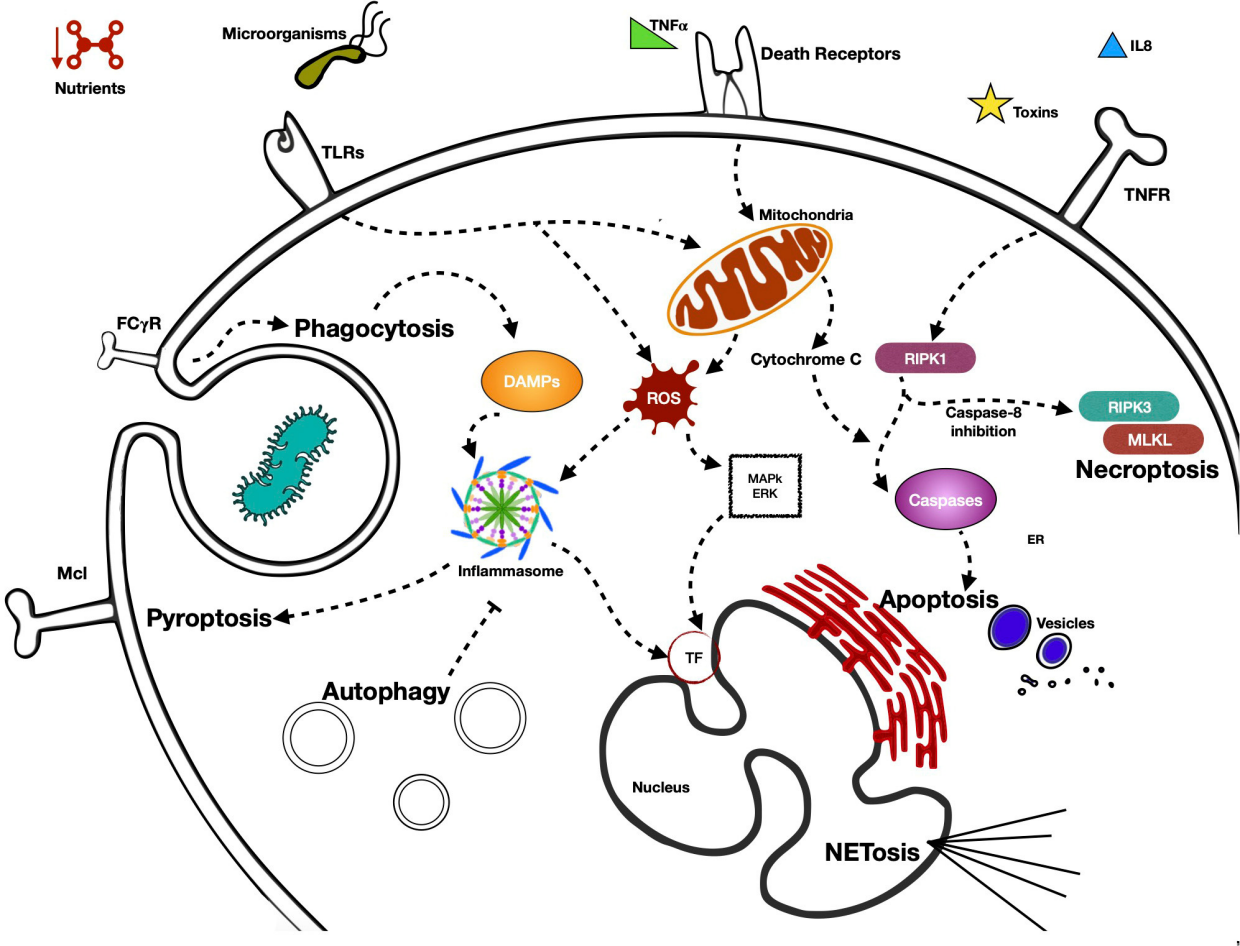
Activation of different type of programmed cell neutrophil death, pathways by neutrophil senses external signals through its PRRs, and triggers effector functions. Neutrophils present in their cellular membrane, a great variety of receptors (pattern recognition receptors, PRR) that are capable of sensoring diverse MAMP (microorganisms associated molecular patterns) and DAMPs (damage-associated molecular patterns). Depending on the nature and intensity of the stimulus received, PRRs are capable of initiating signaling cascades that generally terminate in processes of cell death. These are mainly initiated by proinflammatory proteins, which possess effector functions that lead to the death of neutrophils by means of different mechanisms, such as NETosis, autophagy, necroptosis, or pyroptosis, among others. The receptor that is activated in the processes of cell death is very important, defining as it does the form of cell death that the cell takes. The processes of cell death generally begin with the receptor itself. However, the signaling cascade defines at some point what the fate of the neutrophil will be.

In Figure 1 we summarize the various pathways by which the neutrophil detects external signals through its PRRs and triggers effector functions, in addition to the activation of the different types of programmed cell death and the main signaling molecules involved.

## Neutrophils Death and Disease

Neutrophil cell death has been found to participate in the pathophysiology of various diseases. The elucidation of the mechanisms involved is important to design prophylactic or therapeutic strategies to help patients. Given that great numbers of neutrophils are released daily to the circulation, a precise regulation of neutrophil death is essential to maintain the homeostatic equilibrium in the body. Neutrophils are of great importance in pathogen elimination due to their powerful antimicrobial arsenal. However, many of these microbicidal molecules can also enhance inflammation and tissue injury and can be detrimental for several inflammatory diseases.

### Clinical Implications With Apoptosis and Netosis

#### Apoptosis

In a normal inflammatory process, the apoptotic cells are recognized and removed by macrophages through efferocytosis, so that the inflammation process is regulated. Thus, it is important for neutrophils to undergo spontaneous apoptosis to contribute to the normal resolution of infections or inflammation. Conditions that delay or prevent neutrophil apoptosis result in neutrophilia and this can be a major contributor to chronicity. Gray et al. observed this phenomenon in cystic fibrosis patients, but a similar situation has been seen in many clinical conditions, especially in patients with lung diseases, such as lung cancer (240, 241), asthma (242, 243), chronic obstructive pulmonary disease (COPD) (244, 245), among others. Additionally, the extent of neutrophil apoptosis was found to be inversely proportional to the severity of sepsis in acute respiratory distress syndrome (ARDS). The mean percentage of apoptosis was significantly lower in sepsis-induced ARDS patients compared with uncomplicated sepsis (246).

A delay in neutrophil apoptosis has also been seen in inflammatory bowel disease (IBD) and the induction of apoptosis by the activation of different signaling pathways seems to be a possible solution for this disease. Zhang et al. observed that, in patients with IBD, treatment with TNF-α mAbs induces neutrophil apoptosis leading the resolution of inflammation in the intestinal mucosa (247). In a different study, Rossi et al. induced apoptosis of neutrophils using CDK inhibitors (that induce caspase dependent apoptosis) and found this facilitated the resolution of established neutrophil-dependent inflammation, suggesting this approach could be used to aid in the treatment of chronic inflammatory diseases (248).

On the other hand, a potentiated apoptosis could result in neutropenia and there are many pathological conditions related to this. Neutropenia is primarily associated with severe systemic bacterial infection. However, neutropenia due to excessive apoptosis can be found in systemic lupus erythematosus patients (249), patients undergoing hemodialysis (where apoptosis is related to the deficient biocompatibility of hemodialysis systems) (250), and congenital pathologies, such as myelokathexis, in which myeloid progenitors undergo spontaneous apoptosis due to the down regulated expression of *bcl-x*, an important regulator of apoptosis in hematopoietic cells (251).

#### NETosis

At the end of last century, the discovery of NETosis and its ability to kill bacteria gave rise to a whole new area of research. As the study of this phenomena advanced, it became evident that even though NETs are important effectors in the clearing of bacterial infections, they can also cause tissue injury (252–254). The precise regulation of NET release and the ensuing NETosis, the same as other cell death forms, is essential for maintaining the homeostatic equilibrium in the body. As mentioned before, a large array of factors that can cause inflammation are released during NETosis. Histones, e.g., are able to directly bind and activate platelets, accelerating thrombin production in a mechanism that is mediated by TLR2 and TLR4 (255, 256). In 2007, it was demonstrated in an experimental model of sepsis that TLR4-dependent platelet-neutrophil interactions induced NET formation and that NETs are able to trap circulating *E. coli*, but at the expense of injury to the endothelium and tissue (257).

NETosis has been shown to be involved in many inflammatory processes and diseases ranging from systemic lupus erythematosus (258) to the new COVID-19. Neutrophils increase dramatically in COVID-19 and ARDS: cytokines that induce NETosis are present in patients' lungs and serum during SARS-CoV-2 infection (259–261). In addition, it has been suggested that NETs could be used as a severity marker in COVID-19 (262).

Previous reports have correlated NETs with pulmonary diseases, particularly ARDS. It has been seen that NETs, and in particular certain components like histones, are present in much higher levels in ARDS than in healthy controls, potentiating the severity of the syndrome and patient's mortality (263–266). In addition, NETs from excessive NETosis makes mucus thicker and more viscous, facilitating bacterial infection, which in turn recruits more neutrophils and promotes further production of NETs. Consequently, the respiratory function of patients is progressively affected, increasing the severity of the disease (267).

NETs release by activated neutrophils in circulation can promote thrombosis in veins and arteries (268, 269). It has been shown in thrombosis animal models that coagulation may be enhanced by direct NET-dependent activation of the contact system (256, 270).

Moreover, a correlation has been demonstrated between NETs production and IL-1β and IL-6, associated with a pro-inflammatory state (271, 272) and the relationship between NETs and permanent organ damage in the cardiovascular and renal systems is well-known (273, 274). The potential of NETs to induce tissue damage underlines the importance of considering NETs evaluation in severe COVID-19 patients, it and suggests that targeting NETs may lessen the severe multi-organ consequences of COVID-19 (275).

#### Clinical Implications of Other Types of Neutrophil Death

Apoptosis and NETosis are very important neutrophil death forms in disease. Apoptosis due to the homeostatic regulation that must be accomplished in the body and its relation to neutrophils activity, and NETosis due to its ability to enhance inflammation, contributing to the pathophysiology of several diseases. The contribution of these two types of neutrophil death to disease has received much attention, although other types of neutrophil cell death can also contribute to disease.

Autophagy could participate in disease in a similar way to apoptosis, since enhanced autophagy can result in neutropenia. Olcay et al. showed marked autophagy in neutrophils of patients with congenital dysgranulopoietic neutropenia (CDN), although it cannot be proved that the excess of autophagy may have caused the neutropenia (276).

Neutrophils are the first line of cellular protection against infection, so many of the clinical problems related to dysregulation of their programed cell death is reflected in relation to infection/sepsis diseases.

Pyroptosis is essentially a form of programmed cell death caused by intracellular pathogen infection. Although neutrophils are especially resistant to pyroptosis, it has been demonstrated that they can undergo this form of programmed cell death when there is an absence of a functional NADPH oxidase Nox2 during acute lung infection with *P. aeruginosa* (123), probably to compensate the absence of an antimicrobial pathway. Since neutrophils play an important role in sepsis, it has been suggested that the regulation of neutrophil pyroptosis may positively impact sepsis treatment (277).

Necrosis is usually considered a more pathological form of cell death. When neutrophils die by necrosis, their cytoplasmatic content may cause additional tissue damage or inflammation and it could promote chronic inflammation rather than the resolution of the problem (278, 279). Most of the clinically detrimental effects related to neutrophil necrosis are caused by the virulence of bacteria. This is the case of patients with chronic granulomatous disease, whose neutrophils undergo necrosis after *Burkholderia cenocepacia* infection (280) or the *Haemophilus influenzae* infection, commonly found in patients with COPD (281).

There are some studies that have related chronic inflammation to programmed neutrophil necrosis (282), but there is not much research about pathologies resulting specifically from neutrophil necroptosis. The most known cell types that are correlated with necroptosis-diseases in humans are epithelial liver (270), intestine, and skin cells (68). It was recently found that in patients with neutrophilic diseases, including cutaneous vasculitis, ulcerative colitis, and psoriasis, the migration of neutrophils to infection sites can trigger necroptosis through different adhesion molecules (76).

## Conclusion

The humoral and cellular components of the innate and acquired immune response form a host-defense integrated system, in which numerous cells and molecules function collectively. Cells of the innate immune response are not only effector cells that can attack the invading microbe immediately, but they also influence the type of specific adaptive response that is subsequently developed. In this regard, neutrophils are very important participants in the immunological response. This is not only because they are important effector cells with great microbicidal activity, but also for the role their products play in the initiation of an adaptive immune response. Since the lifespan of neutrophils is short due to a constitutive program for apoptosis, cytokines and other stimuli at inflammatory sites can delay this apoptosis program. On the other hand, once the pathogenic threat is eliminated, neutrophil death and their subsequent clearance by macrophages is extremely important for the resolution of inflammation. Dysregulation of the rate of neutrophils death can have deleterious effects. Accelerated death or neutrophils can lead to inefficient pathogen clearance, but delays in their death can result in exacerbated tissue damage and chronic inflammation. Thus, the study of the mechanisms regulating neutrophil death and survival, both in homeostasis and in inflammatory states, is of great interest.

It is noteworthy that all the mechanisms that trigger the death of neutrophils are important to provide signals that contribute to the initiation of an adaptive response, as well as to the recovery of the homeostasis of the tissue and the organism, which are also possible targets of inflammatory diseases (Tables 1, 2). A better understanding of the mechanisms underlying the cell death of neutrophils will aid in understanding their physiology and will contribute in the search for novel approaches for the management of pathologies such as, cystic fibrosis, asthma, COPD, ARDS, sepsis, IBD, systemic lupus erythematosus, myelokathexis, COVID-19, thrombosis, cutaneous vasculitis, ulcerative colitis, and psoriasis, all of them related with alterations in the death mechanisms of these cells. It will also increase our understanding of inflammation in general, allowing us to identify new factors, molecules, and mechanisms that could have possible medical applications. Therefore, their study and their effects are maintained as a broad field of research.

**Table 1:** Receptors involved in distinct mechanisms of cell death of neutrophils.

**Apoptosis**	**Necroptosis**	**NETosis**	**Autophagy**	**Phagocytosis**
TNFR (201, 219)	Death receptors:	FPR1/2	TLR	FCγR
FAS (APO-1/CD95) (232)	TNFR	FCgR (283)	NOD	
DR3 (APO-3/TRAMP) (201)	TLR	nAChR (284)	NLR	
DR4 (TRAIL-R1) (236)	INFAR	CR3 (285)	CLR	
DR5 (TRAIL-R2(236)		C5aR (when IFN-β is used as a priming cytokine) (286)		
DR6 (201)	Adhesion receptors:			
	CD11b			
	CD15			
	CD18 (62)			

**Table 2:** Sensor proteins and signal cell death in distinct mechanisms of cell death of neutrophils.

**Apoptosis**	**Necrosis**	**Necroptosis**	**Pyroptosis**	**NETosis**
Caspase−2,−3,−6,−7,−8,−9,−10	HIF	RIPK1	Caspase−1,−11	Platelet-neutrophil interaction
Mitochondrial [Ca^2+^] uptake	Nutrients	RIPK3	Inflammasome	
PIDDosome	Temperature	MLKL		
Cythocrome C, ROS	Mechanical stress	NADPH oxidase (via NETosis)		
	Bacteria			

## Author Contributions

EP-F and PÁ-C reviewed the literature and prepared drafts of the manuscript. EO and CM-B assisted in evaluation of the literature and finalizing manuscript for submission. All authors contributed to the article and approved the submitted version.

## Conflict of Interest

The authors declare that the research was conducted in the absence of any commercial or financial relationships that could be construed as a potential conflict of interest.

## References

[1] Kantari C Pederzoli-Ribeil M Witko-Sarsat V. The role of neutrophils and monocytes in innate immunity. Contrib Microbiol. (2008) 15:118–46. 10.1159/000136335 18511859

[2] Cascão R Rosário HS Souto-Carneiro MM Fonseca JE. Neutrophils in rheumatoid arthritis: more than simple final effectors. Autoimmun Rev. (2010) 9:531–5. 10.1016/j.autrev.2009.12.013 20060506

[3] Kuijpers TW Hakkert BC Hart MHL Roos D. Neutrophil migration across monolayers of cytokine-prestimulated endothelial cells: a role for platelet-activating factor and IL-8. J Cell Biol. (1992) 117:565–72. 10.1083/jcb.117.3.565 1315317 PMC2289445

[4] Borregaard N Theilgaard-Mönch K Cowland JB Ståhle M Sørensen OE. Neutrophils and keratinocytes in innate immunity-cooperative actions to provide antimicrobial defense at the right time and place. J Leukoc Biol. (2005) 0.77:439–43. 10.1189/jlb.0704381 15582983

[5] Soehnlein O Lindbom L. Phagocyte partnership during the onset and resolution of inflammation. Nat Rev Immunol. (2010) 10:427–39. 10.1038/nri2779 20498669

[6] Groselj-Grenc M Ihan A Derganc M. Neutrophil and monocyte CD64 and CD163 expression in critically Ill neonates and children with sepsis: comparison of fluorescence intensities and calculated indexes. Mediators Inflamm. (2008) 2008:1–10. 10.1155/2008/202646 18604302 PMC2442385

[7] Futosi K Fodor S Mócsai A. Neutrophil cell surface receptors and their intracellular signal transduction pathways. Int Immunopharmacol. (2013) 17:638–50. 10.1016/j.intimp.2013.06.034 23994464 PMC3827506

[8] Czop JK Puglisi AV Miorandi DZ Austen KF. Perturbation of β-glucan receptors on human neutrophils initiates phagocytosis and leukotriene B4 production. J Immunol. (1998) 141:3170–6. 2844908

[9] Taylor PR Brown GD Reid DM Willment JA Martinez-Pomares L Gordon S. The β-glucan receptor, dectin-1, is predominantly expressed on the surface of cells of the monocyte/macrophage and neutrophil lineages. J Immunol. (2002) 169:3876–82. 10.4049/jimmunol.169.7.3876 12244185

[10] Haziot A Tsuberi BZ Goyert SM. Neutrophil CD14: Biochemical properties and role in the secretion of tumor necrosis factor-α in response to lipopolysaccharide. J Immunol. (1993) 0.150:5556–5565. 7685797

[11] Antal-Szalmas P Strijp JA Weersink AJ Verhoef J Van Kessel KP. Quantitation of surface CD14 on human monocytes and neutrophils. J Leukoc Biol. (1997) 61:721–8. 10.1002/jlb.61.6.721 9201263

[12] Leigh LE Ghebrehiwet B Perera TP Bird IN Strong P Kishore U. C1q-mediated chemotaxis by human neutrophils: involvement of gClqR and G-protein signalling mechanisms. Biochem J. (1998) 330:247–54. 10.1042/bj3300247 9461517 PMC1219134

[13] Zhou MJ Lublin DM Link DC Brown EJ. Distinct tyrosine kinase activation and triton X-100 insolubility upon FcγRII or FcγRIIIB ligation in human polymorphonuclear leukocytes: Implications for immune complex activation of the respiratory burst. J Biol Chem. (1995) 270:13553–60. 10.1074/jbc.270.22.13553 7768958

[14] Coxon A Cullere X Knight S Sethi S Wakelin MW Stavrakis G. FcγRIII mediates neutrophil recruitment to immune complexes: a mechanism for neutrophil accumulation in immune-mediated inflammation. Immunity. (2001) 14:693–704. 10.1016/S1074-7613(01)00150-9 11420040

[15] Selvaraj P Fifadara N Nagarajan S Cimino A Wang G. Functional regulation of human neutrophil Fc γ receptors. Immunol Res. (2004) 29:219–30. 10.1385/IR:29:1-3:219 15181284

[16] Jakus Z Németh T Verbeek JS Mócsai A. Critical but overlapping role of fcγriii and fcγriv in activation of murine neutrophils by immobilized immune complexes. J Immunol. (2008) 180:618–29. 10.4049/jimmunol.180.1.618 18097064 PMC2647079

[17] van Bruggen R Drewniak A Jansen M van Houdt M Roos D Chapel H. Complement receptor 3, not Dectin-1, is the major receptor on human neutrophils for β-glucan-bearing particles. Mol Immunol. (2009) 47:75–81. 10.1016/j.molimm.2009.09.018 19811837

[18] Weckbach LT Gola A Winkelmann M Jakob SM Groesser L Borgolte J. The cytokine midkine supports neutrophil trafficking during acute inflammation by promoting adhesion via β2 integrins (CD11/CD18). Blood. (2014) 123:1887–96. 10.1182/blood-2013-06-510875 24458438

[19] Greenlee-Wacker MC. Clearance of apoptotic neutrophils and resolution of inflammation. Immunol Rev. (2016) 273:357–70. 10.1111/imr.12453 27558346 PMC5000862

[20] Hayashi F Means TK Luster AD. Toll-like receptors stimulate human neutrophil function. Blood. (2003) 102:2660–9. 10.1182/blood-2003-04-1078 12829592

[21] Ekman AK Cardell LO. The expression and function of Nod-like receptors in neutrophils. Immunology. (2010) 130:55–63. 10.1111/j.1365-2567.2009.03212.x 20002790 PMC2855793

[22] Break TJ Jun S Indramohan M Carr KD Sieve AN Berg RE. Extracellular superoxide dismutase inhibits innate immune responses and clearance of an intracellular bacterial infection. J Immunol. (2012) 188:3342–50. 10.4049/jimmunol.1102341 22393157 PMC3311725

[23] Savil JS Wyllie AH Henson JE Walport MJ Henson PM Haslett C. Macrophage phagocytosis of aging neutrophils in inflammation. Programmed cell death in the neutrophil leads to its recognition by macrophages. J Clin Invest. (1989) 83:865–75. 10.1172/JCI113970 2921324 PMC303760

[24] Haslett C Savill JS Whyte MK Stern M Dransfield I Meagher LC. Granulocyte apoptosis and the control of inflammation. Philos Trans R Soc Lond B Biol Sci. (1994) 345:327–33. 10.1098/rstb.1994.0113 7846130

[25] Maianski NA Maianski AN Kuijpers TW Roos D. Apoptosis of neutrophils. Acta Haematol. (2004) 111:56–66. 10.1159/000074486 14646345

[26] Stock W Hoffman R. White blood cells 1: non-malignant disorders. Lancet. (2000) 355:1351–57. 10.1016/S0140-6736(00)02125-5 10776761

[27] Andrès E Maloisel F. Idiosyncratic drug-induced agranulocytosis or acute neutropenia. Curr Opin Hematol. (2008) 15:15–21. 10.1097/MOH.0b013e3282f15fb9 18043241

[28] Akgul C Moulding DA Edwards SW. Molecular control of neutrophil apoptosis. FEBS Lett. (2001) 487:318–22. 10.1016/S0014-5793(00)02324-3 11163351

[29] Scheel-Toellner D Wang K Assi LK Webb PR Craddock RM Salmon M. Clustering of death receptors in lipid rafts initiates neutrophil spontaneous apoptosis. Biochem Soc Trans. (2004) 32:679–81. 10.1042/BST0320679 15493986

[30] Krysko DV Vanden Berghe T D'Herde K Vandenabeele P. Apoptosis and necrosis: detection, discrimination and phagocytosis. Methods. (2008) 44:205–21. 10.1016/j.ymeth.2007.12.001 18314051

[31] Krysko DV Vanden Berghe T Parthoens E D'Herde K Vandenabeele P. Methods for distinguishing apoptotic from necrotic cells and measuring their clearance. Methods Enzymol. (2008) 442:307–41. 10.1016/S0076-6879(08)01416-X 18662577

[32] Geering B Simon HU. Peculiarities of cell death mechanisms in neutrophils. Cell Death Differ. (2011) 18:1457–69. 10.1038/cdd.2011.75 21637292 PMC3178425

[33] Gabelloni ML Trevani AS Sabatté J Geffner J. Mechanisms regulating neutrophil survival and cell death. Semin Immunopathol. (2013) 35:423–37. 10.1007/s00281-013-0364-x 23370701

[34] Whyte MK Meagher LC Macdermot J Haslett C. Impairment of function in aging neutrophils is impairment of function in aging neutrophils is associated with apoptosis. J Immunol. (1993) 150:5124–5134. 8388425

[35] Serhan CN Savill J. Resolution of inflammation: the beginning programs the end. Nat Immunol. (2005) 6:1191–7. 10.1038/ni1276 16369558

[36] Dransfield I Stocks S Haslett C. Regulation of cell adhesion molecule expression and function associated with neutrophil apoptosis. Blood. (1995) 85:3264–73. 10.1182/blood.V85.11.3264.bloodjournal85113264 7538822

[37] Jones J Morgan BP. Apoptosis is associated with reduced expression of complement regulatory molecules, adhesion molecules and other receptors on polymorphonuclear leucocytes: functional relevance and role in inflammation. Immunology. (1995) 86:651–60. 8567034 PMC1384068

[38] Ayub K Hallett MB. Ca2+ influx shutdown during neutrophil apoptosis: importance and possible mechanism. Immunology. (2004) 111:8–12. 10.1111/j.1365-2567.2004.01766.x 14678192 PMC1782384

[39] Yamamoto A Taniuchi S Tsuji S Hasui M Kobayashi Y. Role of reactive oxygen species in neutrophil apoptosis following ingestion of heat-killed *Staphylococcus aureus*. Clin Exp Immunol. (2002) 129:479–84. 10.1046/j.1365-2249.2002.01930.x 12197889 PMC1906464

[40] Medan D Wang L Yang X Dokka S Castranova V Rojanasakul Y. Induction of neutrophil apoptosis and secondary necrosis during endotoxin-induced pulmonary inflammation in mice. J Cell Physiol. (2002) 191:320–6. 10.1002/jcp.10105 12012327

[41] Scannell M Flanagan MB DeStefani A Wynne KJ Cagney G Godson C. Annexin-1 and peptide derivatives are released by apoptotic cells and stimulate phagocytosis of apoptotic neutrophils by macrophages. J Immunol. (2007) 178:4595–605. 10.4049/jimmunol.178.7.4595 17372018

[42] Michlewska S Dransfield I Megson IL Rossi AG. Macrophage phagocytosis of apoptotic neutrophils is critically regulated by the opposing actions of pro-inflammatory and anti-inflammatory agents: key role for TNF-alpha. FASEB J. (2009) 23:844–54. 10.1096/fj.08-121228 18971259

[43] Colotta F Re F Polentarutti N Sozzani S Mantovani A. Modulation of granulocyte survival and programmed cell death by cytokines and bacterial products. Blood. (1992) 80:2012–20. 10.1182/blood.V80.8.2012.2012 1382715

[44] Lee A Whyte MK Haslett C. Inhibition of apoptosis and prolongation of neutrophil functional longevity by inflammatory mediators. J Leukoc Biol. (1993) 54:283–8. 10.1002/jlb.54.4.283 8409750

[45] Kobayashi Y. The role of chemokines in neutrophil biology. Front Biosci. (2008) 13:2400–7. 10.2741/2853 17981721

[46] Simon HU. Neutrophil apoptosis pathways and their modifications in inflammation. Immunol Rev. (2003) 193:101–10. 10.1034/j.1600-065X.2003.00038.x 12752675

[47] Hu Z Sayeed MM. Activation of PI3-kinase/PKB contributes to delay in neutrophil apoptosis after thermal injury. Am J Physiol Cell Physiol. (2005) 5:C1171–8. 10.1152/ajpcell.00312.2004 15625305

[48] Andina N Conus S Schneider EM Fey MF Simon HU. Induction of Bim limits cytokine-mediated prolonged survival of neutrophils. Cell Death Differ. (2009) 16:1248–55. 10.1038/cdd.2009.50 19407828

[49] Kettritz R Xu YX Kerren T Quass P Klein JB Luft FC. Extracellular matrix regulates apoptosis in human neutrophils. Kidney Int. (1999) 55:562–71. 10.1046/j.1523-1755.1999.00280.x 9987080

[50] Ward-Kavanagh LK Lin WW Šedý JR Ware CF. The TNF receptor superfamily in co-stimulating and co-inhibitory responses. Immunity. (2016) 44:1005–19. 10.1016/j.immuni.2016.04.019 27192566 PMC4882112

[51] Beyer K Baukloh AK Stoyanova A Kamphues C Sattler A Kotsch K. Interactions of tumor necrosis factor-related apoptosis-inducing ligand (TRAIL) with the immune system: implications for inflammation and cancer. Cancers. (2019) 11:1161. 10.3390/cancers11081161 31412671 PMC6721490

[52] Sengeløv H Nielsen MH Borregaard N. Separation of human neutrophil plasma membrane from intracellular vesicles containing alkaline phosphatase and NADPH oxidase activity by free flow electrophoresis. J Biol Chem. (1992) 267:14912–7. 10.1016/S0021-9258(18)42127-8 1634531

[53] Simon HU Haj-Yehia A Levi-Schaffer F. Role of reactive oxygen species (ROS) in apoptosis induction. Apoptosis. (2000) 5:415–8. 10.1023/A:1009616228304 11256882

[54] Makni-Maalej K Marzaioli V Boussetta T Belambri SA Gougerot-Pocidalo MA Hurtado-Nedelec M. TLR8, but not TLR7, induces the priming of the NADPH oxidase activation in human neutrophils. J Leukoc Biol. (2015) 97:1081–7. 10.1189/jlb.2A1214-623R 25877926

[55] Buck A Sanchez Klose FP Venkatakrishnan V Khamzeh A Dahlgren C Christenson K. DPI Selectively Inhibits Intracellular NADPH Oxidase Activity in Human Neutrophils. Immunohorizons. (2019) 3:488–97. 10.4049/immunohorizons.1900062 31628159

[56] Douda DN Yip L Khan MA Grasemann H Palaniyar N. Akt is essential to induce NADPH-dependent NETosis and to switch the neutrophil death to apoptosis. Blood. (2014) 123:597–600. 10.1182/blood-2013-09-526707 24458280

[57] Khan MA Farahvash A Douda DN Licht JC Grasemann H Sweezey N. JNK Activation Turns on LPS- and gram-negative bacteria-induced nadph oxidase-dependent suicidal netosis. Sci Rep. (2017) 7:3409. 10.1038/s41598-017-03257-z 28611461 PMC5469795

[58] Nadesalingam A Chen JHK Farahvash A Khan MA. Hypertonic saline suppresses NADPH oxidase-dependent neutrophil extracellular trap formation and promotes apoptosis. Front Immunol. (2018) 9:359. 10.3389/fimmu.2018.00359 29593709 PMC5859219

[59] Azzouz D Khan MA Sweezey N Palaniyar N. Two-in-one: UV radiation simultaneously induces apoptosis and NETosis. Cell Death Discov. 4:51. 10.1038/s41420-018-0048-3 29736268 PMC5919968

[60] Zhang DW Shao J Lin J Zhang N Lu BJ Lin SC. RIP3, an energy metabolism regulator that switches TNF-induced cell death from apoptosis to necrosis. Science. (2009) 325:332–6. 10.1126/science.1172308 19498109

[61] He S Wang L Miao L Wang T Du F Zhao L. Receptor interacting protein kinase-3 determines cellular necrotic response to TNF-α. Cell. (2009) 137:1100–11. 10.1016/j.cell.2009.05.021 19524512

[62] Wang X He Z Liu H Yousefi S Simon HU. Neutrophil necroptosis is triggered by ligation of adhesion molecules following GM-CSF priming. J Immunol. (2016) 197:4090–100. 10.4049/jimmunol.1600051 27815445

[63] Kaiser WJ Sridharan H Huang C Mandal P Upton JW Gough PJ. Toll-like receptor 3-mediated necrosis via TRIF, RIP3, MLKL. J Biol Chem. (2013) 288:31268–79. 10.1074/jbc.M113.462341 24019532 PMC3829437

[64] Thapa RJ Nogusa S Chen P Maki JL Lerro A Andrake M. Interferon-induced RIP1/RIP3-mediated necrosis requires PKR and is licensed by FADD and caspases. Proc Natl Acad Sci USA. (2013) 110:E3109–18. 10.1073/pnas.1301218110 23898178 PMC3746924

[65] Dillon CP Weinlich R Rodriguez DA Cripps JG Quarato G Gurung P. RIPK1 blocks early postnatal lethality mediated by caspase-8 and RIPK3. Cell. (2014) 157:1189–202. 10.1016/j.cell.2014.04.018 24813850 PMC4068710

[66] Benarafa C Simon HU. Role of granule proteases in the life and death of neutrophils. Biochem Biophys Res Commun. (2017) 482:473–81. 10.1016/j.bbrc.2016.11.086 28212734

[67] Upton JW Kaiser WJ Mocarski ES. DAI/ZBP1/DLM-1 complexes with RIP3 to mediate virus-induced programmed necrosis that is targeted by murine cytomegalovirus vIRA. Cell Host Microbe. (2012) 11:290–7. 10.1016/j.chom.2012.01.016 22423968 PMC3531981

[68] Pasparakis M Vandenabeele P. Necroptosis and its role in inflammation. Nature. (2015) 517:311–20. 10.1038/nature14191 25592536

[69] Feoktistova M Geserick P Kellert B Dimitrova DP Langlais C Hupe M. CIAPs block ripoptosome formation, a RIP1/Caspase-8 containing intracellular cell death complex differentially regulated by cflip isoforms. Mol Cell. (2011) 43:449–63. 10.1016/j.molcel.2011.06.011 21737330 PMC3163271

[70] Moulin M Anderton H Voss AK Thomas T Wong WW Bankovacki A. IAPs limit activation of RIP kinases by TNF receptor 1 during development. EMBO J. (2012) 31:1679–91. 10.1038/emboj.2012.18 22327219 PMC3321198

[71] Wong WW Vince JE Lalaoui N Lawlor KE Chau D Bankovacki A. cIAPs and XIAP regulate myelopoiesis through cytokine production in an RIPK1- and RIPK3-dependent manner. Blood. (2014) 123:2562–72. 10.1182/blood-2013-06-510743 24497535

[72] Dondelinger Y Jouan-Lanhouet S Divert T Theatre E Bertin J Gough PJ. NF-κB-Independent role of IKKα/IKKβ in preventing RIPK1 kinase-dependent apoptotic and necroptotic cell death during TNF signaling. Mol Cell. (2015) 60:63–76. 10.1016/j.molcel.2015.07.032 26344099

[73] Wang L Du F Wang X. TNF-α induces two distinct caspase-8 activation pathways. Cell. (2008) 133:693–703. 10.1016/j.cell.2008.03.036 18485876

[74] Sun L Wang H Wang Z He S Chen S Liao D. Mixed lineage kinase domain-like protein mediates necrosis signaling downstream of RIP3 kinase. Cell. (2012) 148:213–27. 10.1016/j.cell.2011.11.031 22265413

[75] Zhao J Jitkaew S Cai Z Choksi S Li Q Luo J. Mixed lineage kinase domain-like is a key receptor interacting protein 3 downstream component of TNF-induced necrosis. Proc Natl Acad Sci USA. (2012) 109:5322–27. 10.1073/pnas.1200012109 22421439 PMC3325682

[76] Wang X Yousefi S Simon H-U. Necroptosis and neutrophil-associated disorders. Cell Death Dis. (2018) 9:111. 10.1038/s41419-017-0058-8 29371616 PMC5833577

[77] Desai J Mulay SR Nakazawa D Anders HJ. Matters of life and death. How neutrophils die or survive along NET release and is “NETosis” = necroptosis? Cell Mol Life Sci. (2016) 73:2211–9. 10.1007/s00018-016-2195-0 27048811 PMC11108262

[78] Golstein P Kroemer G. Cell death by necrosis: towards a molecular definition. Trends Biochem Sci. (2007) 32:37–43. 10.1016/j.tibs.2006.11.001 17141506

[79] Vanden Berghe T Vanlangenakker N Parthoens E Deckers W Devos M Festjens N. Necroptosis, necrosis and secondary necrosis converge on similar cellular disintegration features. Cell Death Differ. (2010) 17:922–30. 10.1038/cdd.2009.184 20010783

[80] Vakifahmetoglu-Norberg H Ouchida AT Norberg E. The role of mitochondria in metabolism and cell death. Biochem Biophys Res Commun. (2017) 482:426–31. 10.1016/j.bbrc.2016.11.088 28212726

[81] Li M Carpio DF Zheng Y Bruzzo P Singh V Ouaaz F. An essential role of the NF- B/toll-like receptor pathway in induction of inflammatory and tissue-repair gene expression by necrotic cells. J Immunol. (2001) 166:7128–35. 10.4049/jimmunol.166.12.7128 11390458

[82] Vanlangenakker N Berghe T Krysko D Festjens N Vandenabeele P. Molecular mechanisms and pathophysiology of necrotic cell death. Curr Mol Med. (2008) 8:207–20. 10.2174/156652408784221306 18473820

[83] Sobolewski A Murray J Bradley JR Cadwallader KA Reed BJ, Chilvers ERAminopeptidase N (CD13) regulates tumor necrosis factor-α-induced apoptosis in human neutrophils. J Biol Chem. (2006) 281:12458–67. 10.1074/jbc.M511277200 16533817

[84] Brinkmann V Reichard U Goosmann C Fauler B Uhlemann Y Weiss DS. Neutrophil extracellular traps kill bacteria. Science. (2004) 303:1532–5. 10.1126/science.1092385 15001782

[85] Yousefi S Simon D Simon HU. Eosinophil extracellular DNA traps: molecular mechanisms and potential roles in disease. Curr Opin Immunol. (2012) 24:736–9. 10.1016/j.coi.2012.08.010 22981682

[86] Fuchs TA Abed U Goosmann C Hurwitz R Schulze I Wahn V. Novel cell death program leads to neutrophil extracellular traps. J Cell Biol. (2007) 176:231–41. 10.1083/jcb.200606027 17210947 PMC2063942

[87] Zawrotniak M Rapala-Kozik M. Neutrophil extracellular traps (NETs) formation and implications. Acta Biochim Pol. (2013) 60:277–84. 10.18388/abp.2013_1983 23819131

[88] Leupold S Büsing P Mas PJ Hart DJ Scrima A. Structural insights into the architecture of the Shigella flexneri virulence factor IcsA/VirG and motifs involved in polar distribution and secretion. J Struct Biol. (2017) 198:19–27. 10.1016/j.jsb.2017.03.003 28268178

[89] Guichon A Hersh D Smith MR Zychlinsky A. Structure-function analysis of the shigella virulence factor IpaB. J Bacteriol. (2001) 183:1269–76. 10.1128/JB.183.4.1269-1276.2001 11157939 PMC95000

[90] Averhoff P Kolbe M Zychlinsky A Weinrauch Y. Single residue determines the specificity of neutrophil elastase for Shigella virulence factors. J Mol Biol. (2008) 377:1053–66. 10.1016/j.jmb.2007.12.034 18295791

[91] Kenny EF Herzig A Krüger R Muth A Mondal S Thompson PR. Diverse stimuli engage different neutrophil extracellular trap pathways. Elife. (2017) 6:1–21. 10.7554/eLife.24437 28574339 PMC5496738

[92] Jorch SK Kubes P. An emerging role for neutrophil extracellular traps in noninfectious disease. Nat Med. (2017) 23:279–87. 10.1038/nm.4294 28267716

[93] Remijsen Q Vanden Berghe T Wirawan E Asselbergh B Parthoens E De Rycke R. Neutrophil extracellular trap cell death requires both autophagy and superoxide generation. Cell Res. (2011) 21:290–304. 10.1038/cr.2010.150 21060338 PMC3193439

[94] Asaga H Senshu T Ishigami A Nakashima K Yamada M. Immunocytochemical localization of peptidylarginine deiminase in human eosinophils and neutrophils. J Leukoc Biol. (2001) 70:46–51. 11435484

[95] Zhou Y An LL Chaerkady R Mittereder N Clarke L Cohen TS. Evidence for a direct link between PAD4-mediated citrullination and the oxidative burst in human neutrophils. Sci Rep. (2018) 8:15228. 10.1038/s41598-018-33385-z 30323221 PMC6189209

[96] Li RHL Ng G Tablin F. Lipopolysaccharide-induced neutrophil extracellular trap formation in canine neutrophils is dependent on histone H3 citrullination by peptidylarginine deiminase. Vet Immunol Immunopathol. (2017) 193–4:29–37. 10.1016/j.vetimm.2017.10.002 29129225

[97] Douda DN Khan MA Grasemann H Palaniyar N. SK3 channel and mitochondrial ROS mediate NADPH oxidase-independent NETosis induced by calcium influx. Proc Natl Acad USA. (2015) 112:2817–22. 10.1073/pnas.1414055112 25730848 PMC4352781

[98] Khan MA D'Ovidio A Tran H Palaniyar N. Anthracyclines suppress both NADPH oxidase- dependent and -independent netosis in human Neutrophils. Cancers (Basel). (2019) 11:1328. 10.3390/cancers11091328 31500300 PMC6770146

[99] Díaz-Godínez C Carrero JC. The state of art of neutrophil extracellular traps in protozoan and helminthic infections. Biosci Rep. (2018) 39:BSR20180916. 10.1042/BSR20180916 30498092 PMC6328873

[100] Papayannopoulos V Metzler KD Hakkim A Zychlinsky A. Neutrophil elastase and myeloperoxidase regulate the formation of neutrophil extracellular traps. J Cell Biol. (2010) 191:677–91. 10.1083/jcb.201006052 20974816 PMC3003309

[101] Urban CF Ermert D Schmid M Abu-Abed U Goosmann C Nacken W. Neutrophil extracellular traps contain calprotectin, a cytosolic protein complex involved in host defense against candida albicans. PLoS Pathog. (2009) 5:e1000639. 10.1371/journal.ppat.1000639 19876394 PMC2763347

[102] Wilkie RP Vissers MCM Dragunow M Hampton MB. A Functional NADPH oxidase prevents caspase involvement in the clearance of phagocytic neutrophils. Infect Immun. (2007) 75:3256–63. 10.1128/IAI.01984-06 17438039 PMC1932946

[103] Hakkim A Fuchs TA Martinez NE Hess S Prinz H Zychlinsky A. Activation of the Raf-MEK-ERK pathway is required for neutrophil extracellular trap formation. Nat Chem Biol. (2011) 7:75–7. 10.1038/nchembio.496 21170021

[104] Carestia A Kaufman T Rivadeneyra L Landoni VI Pozner RG Negrotto S. Mediators and molecular pathways involved in the regulation of neutrophil extracellular trap formation mediated by activated platelets. J Leukoc Biol. (2016) 99:153–62. 10.1189/jlb.3A0415-161R 26320263

[105] Yousefi S Mihalache C Kozlowski E Schmid I Simon HU. Viable neutrophils release mitochondrial DNA to form neutrophil extracellular traps. Cell Death Differ. (2009) 16:1438–44. 10.1038/cdd.2009.96 19609275

[106] Yipp BG Petri B Salina D Jenne CN Scott BN. Infection-induced NETosis is a dynamic process involving neutrophil multitasking in vivo. Nat Med. (2012) 18:1386–93. 10.1038/nm.2847 22922410 PMC4529131

[107] Miao EA Leaf IA Treuting PM Mao DP Dors M Sarkar A. Caspase-1-induced pyroptosis is an innate immune effector mechanism against intracellular bacteria. Nat Immunol. (2010) 11:1136–42. 10.1038/ni.1960 21057511 PMC3058225

[108] Jorgensen I Miao EA. Pyroptotic cell death defends against intracellular pathogens. Immunol Rev. (2015) 265:130–42. 10.1111/imr.12287 25879289 PMC4400865

[109] Schroder K Tschopp J. The inflammasomes. Cell. (2010) 140:821–32. 10.1016/j.cell.2010.01.040 20303873

[110] Proell M Gerlic M Mace PD Reed JC Riedl SJ. The CARD plays a critical role in ASC foci formation and inflammasome signalling. Biochem J. (2013) 449:613–21. 10.1042/BJ20121198 23110696 PMC3966062

[111] Agostini L Martinon F Burns K McDermott MF Hawkins PN Tschopp J. NALP3 forms an IL-1beta-processing inflammasome with increased activity in Muckle-Wells autoinflammatory disorder. Immunity. (2004) 20:319–25. 10.1016/s1074-7613(04)00046-9 15030775

[112] Bürckstümmer T Baumann C Blüml S Dixit E Dürnberger G Jahn H. An orthogonal proteomic-genomic screen identifies AIM2 as a cytoplasmic DNA sensor for the inflammasome. Nat Immunol. (2009) 10:266–72. 10.1038/ni.1702 19158679

[113] de Zoete MR Flavell RA. Detecting “different”: Pyrin senses modified GTPases. Cell Res. (2014) 24:1286–7. 10.1038/cr.2014.101 25091449 PMC4220150

[114] Chavarría-Smith J Vance RE. The NLRP1 inflammasomes. Immunol Rev. (2015) 265:22–34. 10.1111/imr.12283 25879281

[115] Broz P Dixit VM. Inflammasomes: mechanism of assembly, regulation and signalling. Nat Rev Immunol. (2016) 16:407–20. 10.1038/nri.2016.58 27291964

[116] Rathinam VA Fitzgerald KA. Inflammasome complexes: emerging mechanisms and effector functions. Cell. (2016) 165:792–800. 10.1016/j.cell.2016.03.046 27153493 PMC5503689

[117] Fink SL Cookson BT. Caspase-1-dependent pore formation during pyroptosis leads to osmotic lysis of infected host macrophages. Cell Microbiol. (2006) 8:1812–25. 10.1111/j.1462-5822.2006.00751.x 16824040

[118] Bergsbaken T Cookson BT. Macrophage activation redirects Yersinia-infected host cell death from apoptosis to caspase-1-dependent pyroptosis. PLoS Pathog. (2007) 3:1570–82. 10.1371/journal.ppat.0030161 17983266 PMC2048529

[119] Kovacs SB Miao EA. Gasdermins: effectors of pyroptosis *Trends Cell Biol*. (2017) 27:673–84. 10.1016/j.tcb.2017.05.005 PMC5565696 28619472

[120] Fink SL Bergsbaken T Cookson BT. Anthrax lethal toxin and Salmonella elicit the common cell death pathway of caspase-1-dependent pyroptosis via distinct mechanisms. Proc Natl Acad Sci USA. (2008) 105:4312–7. 10.1073/pnas.0707370105 18337499 PMC2393760

[121] Chen KW Groß CJ Sotomayor FV Stacey KJ Tschopp J Sweet MJ. The Neutrophil NLRC4 inflammasome selectively promotes IL-1β Maturation without pyroptosis during acute salmonella challenge. Cell Rep. (2014) 8:570–82. 10.1016/j.celrep.2014.06.028 25043180

[122] Hachim MY Khalil BA Elemam NM Maghazachi AA. Pyroptosis: The missing puzzle among innate and adaptive imm.unity crosstalk. J Leukoc Biol. (2020) 108:323–38. 10.1002/JLB.3MIR0120-625R 32083338

[123] Ryu JC Kim MJ Kwon Y Oh JH Yoon SS Shin SJ. Neutrophil pyroptosis mediates pathology of *P. aeruginosa* lung infection in the absence of the NADPH oxidase NOX2. Mucosal Immunol. (2017) 10:757–74. 10.1038/mi.2016.73 27554297

[124] Kovacs SB Oh C Maltez VI McGlaughon BD Verma A Miao EA. Neutrophil caspase-11 is essential to defend against a cytosol-invasive bacterium. Cell Rep. (2020) 32:107967. 10.1016/j.celrep.2020.107967 32726630 PMC7480168

[125] Dikic I Elazar Z. Mechanism and medical implications of mammalian autophagy. Nat Rev Mol Cell Biol. (2018) 19:349–64. 10.1038/s41580-018-0003-4 29618831

[126] Cuervo AM. Autophagy: many paths to the same end. Mol Cell Biochem. (2004) 263:55–72. 10.1023/B:MCBI.0000041848.57020.57 27520665

[127] Delgado MA Elmaoued RA Davis AS Kyei G Deretic V. Toll-like receptors control autophagy. EMBO J. (2008) 27:1110–21. 10.1038/emboj.2008.31 18337753 PMC2323261

[128] Beertsen W Willenborg M Everts V Zirogianni A Podschun R Schröder B. Impaired phagosomal maturation in neutrophils leads to periodontitis in lysosomal-associated membrane protein-2 knockout mice. J Immunol. (2008) 180:475–82. 10.4049/jimmunol.180.1.475 18097049

[129] Mitroulis I Kourtzelis I Kambas K Rafail S Chrysanthopoulou A Speletas M. Regulation of the autophagic machinery in human neutrophils. Eur J Immunol. (2010) 40:1461–72. 10.1002/eji.200940025 20162553

[130] Pliyev BK Menshikov M. Differential effects of the autophagy inhibitors 3-methyladenine and chloroquine on spontaneous and TNF-α-induced neutrophil apoptosis. Apoptosis. (2012) 17:1050–65. 10.1007/s10495-012-0738-x 22638980

[131] von Gunten S Simon H-U. Autophagic-like cell death in neutrophils induced by autoantibodies. Autophagy. (2007) 3:67–8. 10.4161/auto.3436 17102587

[132] Mihalache CC Yousefi S Conus S Villiger PM Schneider EM Simon HU. Inflammation-associated autophagy-related programmed necrotic death of human neutrophils characterized by organelle fusion events. J Immunol. (2011) 186:6532–42. 10.4049/jimmunol.1004055 21515790

[133] Itohm H Matsuo H Kitamura N Yamamoto S Higuchi T Takematsu H. Enhancement of neutrophil autophagy by an IVIG preparation against multidrug-resistant bacteria as well as drug-sensitive strains. J Leukoc Biol. (2015) 98:107–17. 10.1189/jlb.4A0813-422RRR 25908735 PMC4467167

[134] Tang S Zhang Y Yin SW Gao XJ Shi WW Wang Y. Neutrophil extracellular trap formation is associated with autophagy-related signalling in ANCA-associated vasculitis. Clin Exp Immunol. (2015) 180:408–18. 10.1111/cei.12589 25644394 PMC4449769

[135] Skendros P Mitroulis I Ritis K. Autophagy in neutrophils: from granulopoiesis to neutrophil extracellular traps. Front Cell Dev Biol. (2018) 6:109. 10.3389/fcell.2018.00109 30234114 PMC6131573

[136] Huang J Canadien V Lam GY Steinberg BE Dinauer MC Magalhaes MA. Activation of antibacterial autophagy by NADPH oxidases. Proc Natl Acad Sci USA. (2009) 106:6226–31. 10.1073/pnas.0811045106 19339495 PMC2664152

[137] Chargui A Cesaro A Mimouna S Fareh M Brest P Naquet P. Subversion of autophagy in adherent invasive *Escherichia coli*-infected neutrophils induces inflammation and cell death. PLoS ONE. (2012) 7:e51727. 10.1371/journal.pone.0051727 23272151 PMC3522719

[138] Chargui A El May MV. Autophagy mediates neutrophil responses to bacterial infection. APMIS. (2014) 122:1047–58. 10.1111/apm.12271 24735202

[139] Racanelli AC Kikkers SA Choi AMK Cloonan SM. Autophagy and inflammation in chronic respiratory disease. Autophagy. (2018) 14:221–32. 10.1080/15548627.2017.1389823 29130366 PMC5902194

[140] Germic N Stojkov D Oberson K Yousefi S Simon H-U. Neither eosinophils nor neutrophils require ATG5-dependent autophagy for extracellular DNA trap formation. Immunology. (2017) 152:517–25. 10.1111/imm.12790 28703297 PMC5629432

[141] Ravichandran KS. Beginnings of a good apoptotic meal: the find-me and eat-me signaling pathways. Immunity. (2011) 35:445–55. 10.1016/j.immuni.2011.09.004 22035837 PMC3241945

[142] Bratton DL Henson PM. Neutrophil clearance: when the party is over, clean-up begins. Trends Immunol. (2011) 32:350–7. 10.1016/j.it.2011.04.009 21782511 PMC3151332

[143] Brown GC Neher JJ. Eaten alive! Cell death by primary phagocytosis: “Phagoptosis.” *Trends Biochem Sci*. (2012) 37:325–32. 10.1016/j.tibs.2012.05.002 22682109

[144] Mirnikjoo B Balasubramanian K Schroit AJ. Suicidal membrane repair regulates phosphatidylserine externalization during apoptosis. J Biol Chem. (2009) 284:22512–6. 10.1074/jbc.C109.022913 19561081 PMC2755657

[145] Mirnikjoo B Balasubramanina K Schroit AJ. Mobilization of lysosomal calcium regulates the externalization of phosphatidylserine during Apoptosis. J Biol Chem. (2009) 284:6918–23. 10.1074/jbc.M805288200 19126538 PMC2652277

[146] Jitkaew S Witasp E Zhang S Kagan VE Fadeel B. Induction of caspase- and reactive oxygen species-independent phosphatidylserine externalization in primary human neutrophils: role in macrophage recognition and engulfment. J Leukoc Biol. (2009) 85:427–37. 10.1189/jlb.0408232 19106181 PMC2653945

[147] Park Y Liu G Lorne EF Zhao X Wang J Tsuruta Y. PAI-1 inhibits neutrophil efferocytosis. Proc Natl Acad Sci USA. (2008) 105:11784–9. 10.1073/pnas.0801394105 18689689 PMC2575264

[148] Segal AW Dorling J Coade S. Kinetics of fusion of the cytoplasmic granules with phagocytic vacuoles in human polymorphonuclear leukocytes: biochemical and morphological studies. J Cell Biol. (1980) 85:42–59. 10.1083/jcb.85.1.42 7364874 PMC2110597

[149] Johansson A Jesaitis AJ Lundqvist H Magnusson KE Sjoälin C Karlsson A. Different subcellular localization of cytochrome b and the dormant NADPH-Oxidase in neutrophils and macrophages: effect on the production of reactive oxygen species during phagocytosis. Cell Immunol. (1995) 161:61–71. 10.1006/cimm.1995.1009 7867086

[150] Karlsson A Dahlgren C. Assembly and activation of the neutrophil NADPH oxidase in granule membranes. Antioxid Redox Signal. (2002) 4:49–60. 10.1089/152308602753625852 11970843

[151] Jankowski A Scott CC Grinstein S. Determinants of the phagosomal pH in neutrophils. J Biol Chem. (2002) 277:6059–66. 10.1074/jbc.M110059200 11744729

[152] Huynh KK Grinstein S. Regulation of vacuolar ph and its modulation by some microbial species. Microbiol Mol Biol Rev. (2007) 71:452–62. 10.1128/MMBR.00003-07 17804666 PMC2168644

[153] Coakley RJ Taggart C McElvaney NG O'Neill SJ. Cytosolic pH and the inflammatory microenvironment modulate cell death in human neutrophils after phagocytosis. Blood. (2002) 100:3383–91. 10.1182/blood.V100.9.3383 12384441

[154] Kobayashi SD Braughton KR Palazzolo-Balance AM Kennedy AD Sampaio E Kristosturyan E. Rapid neutrophil destruction following phagocytosis of Staphylococcus aureus. J Innate Immun. (2010) 2:560–75. 10.1159/000317134 20587998 PMC3219502

[155] Surewaard BG de Haas CJ Vervoort F Rigby KM DeLeo FR Otto M. Staphylococcal alpha-phenol soluble modulins contribute to neutrophil lysis after phagocytosis. Cell Microbiol. (2013) 15:1427–37. 10.1111/cmi.12130 23470014 PMC4784422

[156] William R Watson G Redmond HP Wang JH Condron C Bouchier-Hayes D. Neutrophils undergo apoptosis following ingestion of *Escherichia coli*. J Immunol. (1996) 156:3986–92. 8621940

[157] Watson RW Redmond HP Wang JH Bouchier-Hayes D. Bacterial ingestion, tumor necrosis factor-alpha, and heat induce programmed cell death in activated neutrophils. Shock. (1996) 5:47–51. 10.1097/00024382-199601000-00010 8821103

[158] Colamussi ML White MR Crouch E Hartshorn KL. Influenza A virus accelerates neutrophil apoptosis and markedly potentiates apoptotic effects of bacteria. Blood. (1999) 93:2395–403. 10.1182/blood.V93.7.2395 10090951

[159] Bergsbaken T Fink SL Cookson BT. Pyroptosis: host cell death and inflammation. Nat Rev Microbiol. (2009) 7:99–109. 10.1038/nrmicro2070 19148178 PMC2910423

[160] Yoon KW. Dead cell phagocytosis and innate immune checkpoint. BMB Rep. (2017) 50:496–503. 10.5483/BMBRep.2017.50.10.147 28768566 PMC5683818

[161] Hung SL Chiang HH Wu CY Hsu MJ Chen YT. Effects of herpes simplex virus type 1 infection on immune functions of human neutrophils. J Periodontal Res. (2012) 47:635–44. 10.1111/j.1600-0765.2012.01476.x 22471246

[162] Lukaszewicz A-C Gontier G Faivre V Ouanounou I Payen D. Elevated production of radical oxygen species by polymorphonuclear neutrophils in cerebrospinal fluid infection. Ann Intensive Care. (2012) 2:10. 10.1186/2110-5820-2-10 22490368 PMC3359206

[163] Fay AJ Qian X Jan YN Jan LY. SK channels mediate NADPH oxidase-independent reactive oxygen species production and apoptosis in granulocytes. Proc Natl Acad Sci USA. (2006) 103:17548–53. 10.1073/pnas.0607914103 17085590 PMC1634413

[164] Carrichon L Picciocchi A Debeurme F Defendi F Beaumel S Jesaitis AJ. Characterization of superoxide overproduction by the D-Loop Nox4-Nox2 cytochrome b558 in phagocytes-Differential sensitivity to calcium and phosphorylation events. Biochim Biophys Acta. (2011) 1808:78–90. 10.1016/j.bbamem.2010.08.002 20708598 PMC2997891

[165] Pryde JG Walker A Rossi AG Hannah S Haslett C. Temperature-dependent arrest of neutrophil apoptosis. Failure of Bax insertion into mitochondria at 15 °C prevents the release of cytochrome c. J Biol Chem. (2000) 275:33574–84. 10.1074/jbc.M001008200 10896657

[166] Liu CY Takemasa A Liles WC Goodman RB Jonas M Rosen H. Broad-spectrum caspase inhibition paradoxically augments cell death in TNF-alpha -stimulated neutrophils. Blood. (2003) 101:295–304. 10.1182/blood-2001-12-0266 12393619

[167] Maianski NA Geissler J Srinivasula SM Alnemri ES Roos D Kuijpers TW. Functional characterization of mitochondria in neutrophils: a role restricted to apoptosis. Cell Death Differ. (2004) 11:143–53. 10.1038/sj.cdd.4401320 14576767

[168] Kasahara Y Iwai K Yachie A Ohta K Konno A Seki H. Involvement of reactive oxygen intermediates in spontaneous and CD95 (Fas/APO-1)-mediated apoptosis of neutrophils. Blood. (1997) 89:1748–53. 10.1182/blood.V89.5.1748 9057659

[169] Lundqvist-Gustafsson H Bengtsson T. Activation of the granule pool of the NADPH oxidase accelerates apoptosis in human neutrophils. J Leukoc Biol. (1999) 65:196–204. 10.1002/jlb.65.2.196 10088602

[170] Shi T Dansen TB. ROS induced p53 activation: DNA damage, redox signaling or both? Antioxid Redox Signal. (2020) 33:839–59. 10.1089/ars.2020.8074 32151151

[171] Tschopp J Schroder K. NLRP3 inflammasome activation: the convergence of multiple signalling pathways on ROS production? Nat Rev. (2010) 10:210–5. 10.1038/nri2725 20168318

[172] Sorbara MT Girardin SE. Mitochondrial ROS fuel the inflammasome. Cell Res. (2011) 21:558–60. 10.1038/cr.2011.20 21283134 PMC3203657

[173] Kwon YW Masutani H Nakamura H Ishii Y Yodoi J. Redox regulation of cell growth and cell death. Biol Chem. (2003) 384:991–6. 10.1515/BC.2003.111 12956415

[174] Zhang B Hirahashi J Cullere X Mayadas TN. Elucidation of molecular events leading to neutrophil apoptosis following phagocytosis. Cross-talk between caspase 8, reactive oxygen species, and MAPK/ERK activation. J Biol Chem. (2003) 278:28443–54. 10.1074/jbc.M210727200 12736263

[175] Davis RJ. Signal transduction by the JNK group of MAP kinases. Cell. (2000) 103:239–52. 10.1016/S0092-8674(00)00116-1 11057897

[176] Honda S Hirata H Chang L Maeda S Karin M Kamata H. Reactive oxygen species Promote TNFα-Induced death and sustained Jnk activation by inhibiting map kinase phosphatases. Cell. (2005) 120:649–61. 10.1016/j.cell.2004.12.041 15766528

[177] Blomgran R Zheng L Stendahl O. Cathepsin-cleaved Bid promotes apoptosis in human neutrophils via oxidative stress-induced lysosomal membrane permeabilization. J Leukoc Biol. (2007) 81:1213–23. 10.1189/jlb.0506359 17264306

[178] Honda F Kano H Kanegane H Nonoyama S Kim ES Lee SK. The kinase Btk negatively regulates the production of reactive oxygen species and stimulation-induced apoptosis in human neutrophils. Nat Immunol. (2012) 13:369–78. 10.1038/ni.2234 22366891

[179] Blomgran R Zheng L Stendahl O. Uropathogenic *Escherichia coli* TRIGGERS OXYGEN-DEPENDENT APOPTOSIS IN HUMAN NEUTROPHILS THROUGH THE COOPERATIVE EFFECT OF TYPE 1 FIMBRIAE AND LIPOPOLYSACCHARIDE. Infect Immun. (2004) 72:4570–8. 10.1128/IAI.72.8.4570-4578.2004 15271917 PMC470702

[180] Ward C Wong TH Murray J Rahman I Haslett C Chilvers ER. Induction of human neutrophil apoptosis by nitric oxide donors: evidence for a caspase-dependent, cyclic-GMP-independent, mechanism. Biochem Pharmacol. (2000) 59:305–14. 10.1016/S0006-2952(99)00329-9 10609560

[181] Blaylock MG Cuthbertson BH Galley HF Ferguson NR Webster NR. The effect of nitric oxide and peroxynitrite on apoptosis in human polymorphonuclear leukocytes. Free Radic Biol Med. (1998) 25:748–52. 10.1016/S0891-5849(98)00108-7 9801076

[182] Fortenberry JD Owens ML Brown MR Atkinson D Brown LA. Exogenous nitric oxide enhances neutrophil cell death and dna fragmentation. Am J Respir Cell Mol Biol. (1998) 18:421–8. 10.1165/ajrcmb.18.3.2875 9490660

[183] Taylor EL Megson IL Haslett C Rossi AG. Nitric oxide: a key regulator of myeloid inflammatory cell apoptosis. Cell Death Differ. (2003) 10:418–30. 10.1038/sj.cdd.4401152 12719719

[184] Taylor EL Rossi AG Shaw CA Dal Rio FP Haslett C Megson IL. GEA 3162 decomposes to co-generate nitric oxide and superoxide and induces apoptosis in human neutrophils via a peroxynitrite-dependent mechanism. Br J Pharmacol. (2004) 143:179–85. 10.1038/sj.bjp.0705909 15289284 PMC1575270

[185] Johansson AC Appelqvist H Nilsson C Kågedal K Roberg K Öllinger K. Regulation of apoptosis-associated lysosomal membrane permeabilization. Apoptosis. (2010) 15:527–40. 10.1007/s10495-009-0452-5 20077016 PMC2850995

[186] Earnshaw WC Martins LM Kaufmann SH. Mammalian caspases: structure, activation, substrates, and functions during apoptosis. Annu Rev Biochem. (1999) 68:383–424. 10.1146/annurev.biochem.68.1.383 10872455

[187] Kaufmann SH Kottke TJ Martins LM Henzing AJ Earnshaw WC. Analysis of Caspase Activation During Apoptosis. Curr Protoc Cell Biol. (2001) 11:18.2.1–29. 10.1002/0471143030.cb1802s11 18228341

[188] Aref S Abdullah D Fouda M El Menshawy N Azmy E Bassam A. Neutrophil apoptosis in neutropenic patients with hepatitis c infection: role of caspases 3, 10, and GM-CSF. Indian J Hematol Blood Transfus. (2011) 27:81–7. 10.1007/s12288-011-0067-1 22654297 PMC3136665

[189] Pérez-Figueroa E Torres J Sánchez-Zauco N Contreras-Ramos A Alvarez-Arellano L Pérez-Figueroa E. Activation of NLRP3 inflammasome in human neutrophils by Helicobacter pylori infection. Innate Immun. (2016) 22:103–12. 10.1177/1753425915619475 26610398

[190] Smith MA Schnellmann RG. Calpains, mitochondria, and apoptosis. Cardiovasc Res. (2012) 96:32–7. 10.1093/cvr/cvs163 22581845 PMC3444233

[191] Goll DE Thompson VF Li H Wei W Cong J. The calpain system. Physiol Rev. (2003) 83:731–801. 10.1152/physrev.00029.2002 12843408

[192] Altznauer F Conus S Cavalli A Folkers G Simon HU. Calpain-1 regulates bax and subsequent smac-dependent caspase-3 activation in neutrophil apoptosis. J Biol Chem. (2004) 279:5947–57. 10.1074/jbc.M308576200 14612448

[193] Wiemer AJ Lokuta MA Surfus JC Wernimont SA Huttenlocher A. Calpain inhibition impairs TNF-α-mediated neutrophil adhesion, arrest and oxidative burst. Mol Immunol. (2010) 47:894–902. 10.1016/j.molimm.2009.10.002 19889458 PMC2814964

[194] Fujita H Kato T Watanabe N Takahashi T Kitagawa S. Calpain inhibitors stimulate phagocyte functions via activation of human formyl peptide receptors. Arch Biochem Biophys. (2011) 513:51–60. 10.1016/j.abb.2011.06.007 21723247

[195] Maianski NA Roos D Kuijpers TW. Bid truncation, bid/bax targeting to the mitochondria, and caspase activation associated with neutrophil apoptosis are inhibited by granulocyte colony-stimulating factor. J Immunol. (2004) 172:7024–30. 10.4049/jimmunol.172.11.7024 15153524

[196] Murphy BM O'Neill AJ Adrain C Watson RW Martin SJ. The apoptosome pathway to caspase activation in primary human neutrophils exhibits dramatically reduced requirements for cytochrome c. J Exp Med. (2003) 197:625–32. 10.1084/jem.20021862 12615903 PMC2193828

[197] Fossati G Moulding DA Spiller DG Moots RJ White MR Edwards SW. The mitochondrial network of human neutrophils: role in chemotaxis, phagocytosis, respiratory burst activation, and commitment to apoptosis. J Immunol. (2003) 170:1964–72. 10.4049/jimmunol.170.4.1964 12574365

[198] Zhang J He J Xia J Chen Z Chen X. Delayed apoptosis by neutrophils from COPD patients is associated with altered bak, bcl-xl, and mcl-1 mRNA expression. Diagn Pathol. 7:65. 10.1186/1746-1596-7-65 22686245 PMC3488503

[199] Cowburn AS Summers C Dunmore BJ Farahi N Hayhoe RP Print CG. Granulocyte/macrophage colony–stimulating factor causes a paradoxical increase in the bh3-only pro-apoptotic protein bim in human neutrophils. Am J Respir Cell Mol Biol. (2011) 44:879–87. 10.1165/rcmb.2010-0101OC 20705940 PMC4373550

[200] Moulding DA Quayle JA Hart CA Edwards SW. Mcl-1 expression in human neutrophils: regulation by cytokines and correlation with cell survival. Blood. (1998) 92:2495–502. 10.1182/blood.V92.7.2495.2495_2495_2502 9746790

[201] Akgul C Edwards SW. Regulation of neutrophil apoptosis via death receptors. Cell Mol Life Sci. (2003) 60:2402–8. 10.1007/s00018-003-3110-z 14625685 PMC11146070

[202] O'Neill AJ Doyle BT Molloy E Watson C Phelan D Greenan MC. Gene expression profile of inflammatory neutrophils: alterations in the inhibitors of apoptosis proteins during spontaneous and delayed apoptosis. Shock. (2004) 21:512–8. 10.1097/01.shk.0000123512.13212.ca 15167679

[203] Sakamoto E. Type I and type II interferons delay human neutrophil apoptosis via activation of STAT3 and up-regulation of cellular inhibitor of apoptosis 2. J Leukoc Biol. (2005) 78:301–9. 10.1189/jlb.1104690 15845643

[204] François S El Benna J Dang PM Pedruzzi E Gougerot-Pocidalo MA Elbim C. Inhibition of neutrophil apoptosis by tlr agonists in whole blood: involvement of the phosphoinositide 3-kinase/akt and nf-κb signaling pathways, leading to increased levels of mcl-1, a1, and phosphorylated Bad. J Immunol. (2005) 174:3633–42. 10.4049/jimmunol.174.6.3633 15749901

[205] Tsukahara Y Lian Z Zhang X Whitney C Kluger Y Tuck D. Gene expression in human neutrophils during activation and priming by bacterial lipopolysaccharide. J Cell Biochem. (2003) 89:848–61. 10.1002/jcb.10526 12858349

[206] Wei S Liu JH Epling-Burnette PK Gamero AM Ussery D Pearson EW. Critical role of Lyn kinase in inhibition of neutrophil apoptosis by granulocyte-macrophage colony-stimulating factor. J Immunol. (1996) 157:5155–62. 8943427

[207] Yasui K Sekiguchi Y Ichikawa M Nagumo H Yamazaki T. Granulocyte macrophage-colony stimulating factor delays neutrophil apoptosis and primes its function through Ia-type phosphoinositide 3-kinase. J Leukoc Biol. (2002) 72:1020–6. 10.1189/jlb.72.5.1020 12429725

[208] Kobayashi SD. Spontaneous neutrophil apoptosis and regulation of cell survival by granulocyte macrophage-colony stimulating factor. J Leukoc Biol. (2005) 78:1408–18. 10.1189/jlb.0605289 16204629

[209] Maianski NA. Granulocyte colony-stimulating factor inhibits the mitochondria-dependent activation of caspase-3 in neutrophils. Blood. (2002) 99:672–9. 10.1182/blood.V99.2.672 11781253

[210] Bruno A Conus S Schmid I Simon H-U. Apoptotic pathways are inhibited by leptin receptor activation in neutrophils. J Immunol. (2014) 174:8090–6. 10.4049/jimmunol.174.12.8090 15944317

[211] El Kebir D József L Khreiss T Filep JG. Inhibition of K+ efflux prevents mitochondrial dysfunction, and suppresses caspase-3-, apoptosis-inducing factor-, and endonuclease G-mediated constitutive apoptosis in human neutrophils. Cell Signal. (2006) 18:2302–13. 10.1016/j.cellsig.2006.05.013 16806822

[212] Coxon A Tang T Mayadas TN. Cytokine-activated endothelial cells delay neutrophil apoptosis in vitro and in vivo. J Exp Med. (1999) 190:923–34. 10.1084/jem.190.7.923 10510082 PMC2195653

[213] Ross EA Douglas MR Wong SH Ross EJ Curnow SJ Nash GB. Interaction between integrin α9β1 and vascular cell adhesion molecule-1 (VCAM-1) inhibits neutrophil apoptosis. Blood. (2006) 107:1178–83. 10.1182/blood-2005-07-2692 16223772 PMC3132455

[214] Costantini C Micheletti A Calzetti F Perbellini O Pizzolo G Cassatella MA. Neutrophil activation and survival are modulated by interaction with NK cells. Int Immunol. (2010) 22:827–38. 10.1093/intimm/dxq434 20739460

[215] Bae HB Zmijewski JW Deshane JS Zhi D Thompson LC Peterson CB. Vitronectin inhibits neutrophil apoptosis through activation of integrin-associated signaling pathways. Am J Respir Cell Mol Biol. (2012) 46:790–6. 10.1165/rcmb.2011-0187OC 22281987 PMC3380283

[216] Zmijewski JW Bae HB Deshane JS Peterson CB Chaplin DD Abraham E. Inhibition of neutrophil apoptosis by PAI-1. Am J Physiol Cell Mol Physiol. (2011) 301:L247–54. 10.1152/ajplung.00075.2011 21622848 PMC3154633

[217] Van Den Berg JM Weyer S Roos D Kuijpers TW Weening JJ. Divergent effects of tumor necrosis factor α on apoptosis of human neutrophils. J Leukoc Biol. (2001) 69:467–73. 10.1189/jlb.69.3.467 11261795

[218] Wright HL Chikura B Bucknall RC Moots RJ Edwards SW. Changes in expression of membrane TNF, NF-κB activation and neutrophil apoptosis during active and resolved inflammation. Ann Rheum Dis. (2011) 70:537–43. 10.1136/ard.2010.138065 21109521

[219] Dyugovskaya L Polyakov A Ginsberg D Lavie P Lavie L. Molecular pathways of spontaneous and TNF-α-mediated neutrophil apoptosis under intermittent hypoxia. Am J Respir Cell Mol Biol. (2011) 45:154–62. 10.1165/rcmb.2010-0025OC 20870895

[220] Maianski NA Roos D Kuijpers TW. Tumor necrosis factor α induces a caspase-independent death pathway in human neutrophils. Blood. (2003) 101:1987–95. 10.1182/blood-2002-02-0522 12393608

[221] Kilpatrick LE Lee JY Haines KM Campbell DE Sullivan KE Korchak HM. A role for PKC-δ and PI 3-kinase in TNF-α-mediated antiapoptotic signaling in the human neutrophil. Am J Physiol Cell Physiol. (2003) 283:C48–57. 10.1152/ajpcell.00385.2001 12055072

[222] Dunican AL Leuenroth SJ Grutkoski P Ayala A Simms HH. TNFα-induced suppression of PMN apoptosis is mediated through interleukin-8 production. Shock. (2000) 14:284–9. 10.1097/00024382-200014030-00007 11028544

[223] Cowburn AS Deighton J Walmsley SR Chilvers ER. The survival effect of TNF-α in human neutrophils is mediated via NF-κB-dependent IL-8 release. Eur J Immunol. (2004) 34:1733–43. 10.1002/eji.200425091 15162444

[224] Renshaw SA Timmons SJ Eaton V Usher LR Akil M Bingle CD. Inflammatory neutrophils retain susceptibility to apoptosis mediated via the Fas death receptor. J Leukoc Biol. (2000) 67:662–8. 10.1002/jlb.67.5.662 10811006

[225] Sharma R Sharma A Dwivedi S Zimniak P Awasthi S Awasthi YC. 4-hydroxynonenal self-limits fas-mediated DISC-independent apoptosis by promoting export of daxx from the nucleus to the cytosol and its binding to fas. Biochemistry. (2008) 47:143–56. 10.1021/bi701559f 18069800 PMC2564820

[226] Wajant H Pfizenmaier K Scheurich P. Tumor necrosis factor signaling. Cell Death Differ. (2003) 10:45–65. 10.1038/sj.cdd.4401189 12655295

[227] Harper N Farrow SN Kaptein A Cohen GM MacFarlane M. Modulation of tumor necrosis factor apoptosis-inducing ligand- induced NF-kappa B activation by inhibition of apical caspases. J Biol Chem. (2001) 276:34743–52. 10.1074/jbc.M105693200 11461927

[228] Xie P. TRAF molecules in cell signaling and in human diseases. J Mol Signal. (2013) 8:7. 10.1186/1750-2187-8-7 23758787 PMC3697994

[229] Watson RW O'Neill A Brannigan AE Coffey R Marshall JC Brady HR. Regulation of Fas antibody induced neutrophil apoptosis is both caspase and mitochondrial dependent. FEBS. (1999) 453:67–71. 10.1016/S0014-5793(99)00688-2 10403377

[230] Croker BA O'Donnell JA Nowell CJ Metcalf D Dewson G Campbell KJ. Fas-mediated neutrophil apoptosis is accelerated by Bid, Bak, and Bax and inhibited by Bcl-2 and Mcl-1. Proc Natl Acad Sci USA. (2011) 108:13135–40. 10.1073/pnas.1110358108 21768356 PMC3156212

[231] Fecho K Cohen PL. Fas ligand (gld) and Fas (lpr) deficient mice do not show alterations in the extravasation or apoptosis of inflammatory neutrophils. J Leukoc Biol. (1998) 64:373–83. 10.1002/jlb.64.3.373 9738665

[232] Villunger A O'Reilly LA Holler N Adams J Strasser A. FAS Ligand, Bcl-2, granulocyte colony-stimulating factor, and p38 mitogen-activated protein kinase. J Exp Med. (2000) 192:647–58. 10.1084/jem.192.5.647 10974031 PMC2193264

[233] Brown SB Savill J. Phagocytosis triggers macrophage release of Fas ligand and induces apoptosis of bystander leukocytes. J Immunol. (1999) 162:480–5. 9886423

[234] Kamohara H Matsuyama W Shimozato O Abe K Galligan C Hashimoto S. Regulation of tumour necrosis factor-related apoptosis-inducing ligand (TRAIL) and TRAIL receptor expression in human neutrophils. Immunology. (2004) 111:186–94. 10.1111/j.0019-2805.2003.01794.x 15027904 PMC1782413

[235] Koga Y Matsuzaki A Suminoe A Hattori H Hara T. Neutrophil-Derived TNF-Related Apoptosis-Inducing Ligand (TRAIL): a novel mechanism of antitumor effect by neutrophils. Cancer Res. (2004) 64:1037–43. 10.1158/0008-5472.CAN-03-1808 14871835

[236] Renshaw SA Parmar JS Singleton V Rowe SJ Dockrell DH Dower SK. Acceleration of human neutrophil apoptosis by TRAIL. J Immunol. (2003) 170:1027–33. 10.4049/jimmunol.170.2.1027 12517970

[237] Lum JJ Bren G McClure R Badley AD. Elimination of senescent neutrophils by TNF-related apopotosis-inducing ligand. J Immunol. (2005) 175:1232–8. 10.4049/jimmunol.175.2.1232 16002727

[238] McGrath EE Marriott HM Lawrie A Francis SE Sabroe I Renshaw SA. TNF-related apoptosis-inducing ligand (TRAIL) regulates inflammatory neutrophil apoptosis and enhances resolution of inflammation. J Leukoc Biol. (2011) 90:855–65. 10.1189/jlb.0211062 21562052 PMC3644175

[239] Simons MP Nauseef WM Griffith TS. Neutrophils and TRAIL: insights into BCG immunotherapy for bladder cancer. Immunol Res. (2007) 39:79–93. 10.1007/s12026-007-0084-1 17917057

[240] Gray RD Hardisty G Regan KH Smith M Robb CT Duffin R. Delayed neutrophil apoptosis enhances NET formation in cystic fibrosis. Thorax. (2018) 73:134–44. 10.1136/thoraxjnl-2017-210134 28916704 PMC5771859

[241] Barrera L Montes-Servín E Hernandez-Martinez JM García-Vicente MLÁ Montes-Servín E Herrera-Martínez M. CD47 overexpression is associated with decreased neutrophil apoptosis/phagocytosis and poor prognosis in non-small-cell lung cancer patients. Br J Cancer. (2017) 117:385–97. 10.1038/bjc.2017.173 28632731 PMC5537491

[242] Uddin M Nong G Ward J Seumois G Prince LR Wilson SJ. Prosurvival activity for airway neutrophils in severe asthma. Thorax. (2010) 65:684–9. 10.1136/thx.2009.120741 20685741

[243] Grunwell JR Stephenson ST Tirouvanziam R Brown LAS Brown MR Fitzpatrick AM. Children with neutrophil-predominant severe asthma have proinflammatory neutrophils with enhanced survival and impaired clearance. J Allergy Clin Immunol Pract. (2019) 7:516–525.e6. 10.1016/j.jaip.2018.08.024 30193935 PMC6363859

[244] Quint JK Wedzicha JA. The neutrophil in chronic obstructive pulmonary disease. J Allergy Clin Immunol. (2007) 119:1065–71. 10.1016/j.jaci.2006.12.640 17270263

[245] Pletz MW Ioanas M de Roux A Burkhardt O Lode H. Reduced spontaneous apoptosis in peripheral blood neutrophils during exacerbation of COPD. Eur Respir J. (2004) 532–7. 10.1183/09031936.04.00089004 15083750

[246] Fialkow L Fochesatto Filho L Bozzetti MC Milani AR Rodrigues Filho EM Ladniuk RM. Neutrophil apoptosis: a marker of disease severity in sepsis and sepsis-induced acute respiratory distress syndrome. Crit Care. (2006) 10:R155. 10.1186/cc5090 17092345 PMC1794458

[247] Zhang C Shu W Zhou G Lin J Chu F Wu H. Anti-TNF-α therapy suppresses proinflammatory activities of mucosal neutrophils in inflammatory bowel disease. Mediators Inflamm. (2018) 2018:3021. 10.1155/2018/3021863 30595666 PMC6282128

[248] Rossi AG Sawatzky DA Walker A Ward C Sheldrake TA Riley NA. Cyclin-dependent kinase inhibitors enhance the resolution of inflammation by promoting inflammatory cell apoptosis. Nat Med. (2006) 12:1434. 10.1038/nm1206-1434 16951685

[249] Courtney PA Crockard AD Williamson K Irvine AE Kennedy RJ Bell AL. Increased apoptotic peripheral blood neutrophils in systemic lupus erythematosus: relations with disease activity, antibodies to double stranded DNA. and neutropenia. Ann Rheum Dis. (1999) 58:309–14. 10.1136/ard.58.5.309 10225817 PMC1752888

[250] Fukushi T Yamamoto T Yoshida M Fujikura E Miyazaki M Nakayama M. Enhanced neutrophil apoptosis accompanying myeloperoxidase release during hemodialysis. Sci Rep. (2020) 10:21747. 10.1038/s41598-020-78742-z 33303892 PMC7728788

[251] Aprikyan AA Liles WC Park JR Jonas M Chi EY Dale DC. Myelokathexis, a congenital disorder of severe neutropenia characterized by accelerated apoptosis and defective expression of bcl-x in neutrophil precursors. Blood. (2000) 95:320–7. 10.1182/blood.V95.1.320 10607719

[252] McDonald B Urrutia R Yipp BG Jenne CN Kubes P. Intravascular neutrophil extracellular traps capture bacteria from the bloodstream during sepsis. Cell Host Microbe. (2012) 12:324–33. 10.1016/j.chom.2012.06.011 22980329

[253] Tanaka K Koike Y Shimura T Okigami M Ide S Toiyama Y. In vivo characterization of neutrophil extracellular traps in various organs of a murine sepsis model. PLoS ONE. (2014) 9:e111888. 10.1371/journal.pone.0111888 25372699 PMC4221155

[254] Sakurai K Miyashita T Okazaki M Yamaguchi T Ohbatake Y Nakanuma S. Role for Neutrophil Extracellular Traps (NETs) and platelet aggregation in early sepsis-induced hepatic dysfunction. In vivo. (2017) 31:1051–8. 10.21873/invivo.11169 29102925 PMC5756631

[255] Semeraro F Ammollo CT Morrissey JH Dale GL Friese P Esmon NL. Extracellular histones promote thrombin generation through platelet-dependent mechanisms: involvement of platelet TLR2 and TLR4. Blood. (2011) 118:1952–61. 10.1182/blood-2011-03-343061 21673343 PMC3158722

[256] Noubouossie DF Reeves BN Strahl BD Key NS. Neutrophils: back in the thrombosis spotlight. Blood. (2019) 133:2186–97. 10.1182/blood-2018-10-862243 30898858 PMC7218731

[257] Clark SR Ma AC Tavener SA McDonald B Goodarzi Z Kelly MM. Platelet TLR4 activates neutrophil extracellular traps to ensnare bacteria in septic blood. Nat Med. (2007) 13:463–9. 10.1038/nm1565 17384648

[258] Hakkim A Fürnrohr BG Amann K Laube B Abed UA Brinkmann V. Impairment of neutrophil extracellular trap degradation is associated with lupus nephritis. Proc Natl Acad Sci USA. (2010) 107:9813–8. 10.1073/pnas.0909927107 20439745 PMC2906830

[259] Tomar B Anders HJ Desai J Mulay SR. Neutrophils and neutrophil extracellular traps drive necroinflammation in COVID-19. Cells. (2020) 9:1383. 10.3390/cells9061383 32498376 PMC7348784

[260] Veras FP Pontelli MC Silva CM Toller-Kawahisa JE de Lima M Nascimento DC. SARS-CoV-2-triggered neutrophil extracellular traps mediate COVID-19 pathology. J Exp Med. (2020) 217:e20201129. 10.1084/jem.20201129 32926098 PMC7488868

[261] Wang J Li Q Yin Y Zhang Y Cao Y Lin X. Excessive neutrophils and neutrophil extracellular traps in COVID-19. Front Immunol. (2020) 11:2063. 10.3389/fimmu.2020.02063 33013872 PMC7461898

[262] Zuo Y Yalavarthi S Shi H Gockman K Zuo M Madison JA. Neutrophil extracellular traps (NETs) as markers of disease severity in COVID-19. medRxiv [Preprint]. (2020). 10.1101/2020.04.09.20059626 32511633 PMC7276989

[263] Chaput C Zychlinsky A. Sepsis: the dark side of histones. Nat Med. (2009) 15:1245–46. 10.1038/nm1109-1245 19893552

[264] Xu Z Huang Y Mao P Zhang J Li Y. Sepsis and ARDS: the dark side of histones. Mediators Inflamm. (2009) 2015:205054. 10.1155/2015/205054 26609197 PMC4644547

[265] Lv X Wen T Song J Xie D Wu L Jiang X. Extracellular histones are clinically relevant mediators in the pathogenesis of acute respiratory distress syndrome. Respir Res. (2017) 18:165. 10.1186/s12931-017-0651-5 28865478 PMC5581408

[266] Wygrecka M Kosanovic D Wujak L Reppe K Henneke I Frey H. Antihistone Properties of C1 esterase inhibitor protect against lung injury. Am J Respir Crit Care Med. (2017)196:186–99. 10.1164/rccm.201604-0712OC 28005404

[267] Manzenreiter R Kienberger F Marcos V Schilcher K Krautgartner WD Obermayer A. Ultrastructural characterization of cystic fibrosis sputum using atomic force and scanning electron microscopy. J Cyst Fibros. (2012) 11:84–92. 10.1016/j.jcf.2011.09.008 21996135

[268] Fuchs TA Brill A Wagner DD. Neutrophil extracellular trap (NET) impact on deep vein thrombosis. Arterioscler Thromb Vasc Biol. (2012) 32:1777–83. 10.1161/ATVBAHA.111.242859 22652600 PMC3495595

[269] Laridan E Martinod K De Meyer SF. Neutrophil extracellular traps in arterial and venous thrombosis. Semin Thromb Hemost. (2019) 45:86–93. 10.1055/s-0038-1677040 30634198

[270] Zhang Z Xie G Liang L Liu H Pan J Cheng H. RIPK3-mediated necroptosis and neutrophil infiltration are associated with poor prognosis in patients with alcoholic cirrhosis. J Immunol Res. (2018) 2018:1509851. 10.1155/2018/1509851 30596105 PMC6286738

[271] Calabrese LH Rose-John S. IL-6 biology: implications for clinical targeting in rheumatic disease. Nat Rev Rheumatol. (2014) 10:720–7. 10.1038/nrrheum.2014.127 25136784

[272] Mehta P McAuley DF Brown M Sanchez E Tattersall RS Manson JJ HLH. COVID-19: consider cytokine storm syndromes and immunosuppression. Lancet. (2020) 395:1033–4. 10.1016/S0140-6736(20)30628-0 32192578 PMC7270045

[273] Kessenbrock K Krumbholz M Schönermarck U Back W Gross WL Werb Z. Netting neutrophils in autoimmune small-vessel vasculitis. Nat Med. (2009) 15:623–5. 10.1038/nm.1959 19448636 PMC2760083

[274] Papayannopoulos V. Neutrophil extracellular traps in immunity and disease. Nat Rev Immunol. (2018) 18:134–47. 10.1038/nri.2017.105 28990587

[275] Ouwendijk WJD Raadsen MP van Kampen JJA Verdijk RM von der Thusen JH Guo L. Neutrophil extracellular traps persist at high levels in the lower respiratory tract of critically ill COVID-19 patients. J Infect Dis. (2021). 10.1093/infdis/jiab053. [Epub ahead of print]. 33507309 PMC7928833

[276] Olcay L Yetgin S Erdemli E Germeshausen M Aktas D Büyükaşik Y. Congenital dysgranulopoietic neutropenia. Pediatr Blood Cancer. (2008) 50:115–9. 10.1002/pbc.20877 16652351

[277] Liu L Sun B. Neutrophil pyroptosis: new perspectives on sepsis. Cell Mol Life Sci. (2019) 76:2031−42. 10.1007/s00018-019-03060-1 30877336 PMC11105444

[278] Savill JS Henson PM Haslett C. Phagocytosis of aged human neutrophils by macrophages is mediated by a novel “charge-sensitive” recognition mechanism. J Clin Invest. (1989) 84:1518–27. 10.1172/JCI114328 2553775 PMC304017

[279] Rydell-Törmänen K Uller L Erjefält JS. Direct evidence of secondary necrosis of neutrophils during intense lung inflammation. Eur Respir J. (2006) 28:268–74. 10.1183/09031936.06.00126905 16510453

[280] Bylund J Campsall PA Ma RC Conway BA Speert DP. Burkholderia cenocepacia induces neutrophil necrosis in chronic granulomatous disease. J Immunol. (2005) 174:3562–9. 10.4049/jimmunol.174.6.3562 15749893

[281] Naylor EJ Bakstad D Biffen M Thong B Calverley P Scott S. Haemophilus influenzae induces neutrophil necrosis: a role in chronic obstructive pulmonary disease? Am J Respir Cell Mol Biol. (2007) 37:135–43. 10.1165/rcmb.2006-0375OC 17363778

[282] Han J Zhong CQ Zhang DW. Programmed necrosis: backup to and competitor with apoptosis in the immune system. Nat Immunol. (2011) 12:1143–9. 10.1038/ni.2159 22089220

[283] Alemán OR Mora N Cortes-Vieyra R Uribe-Querol E Rosales C. Differential use of human neutrophil fc γ receptors for inducing neutrophil extracellular trap formation. J Immunol Res. (2016) 2016:2908034. 10.1155/2016/2908034 27034964 PMC4806689

[284] Hosseinzadeh A Thompson PR Segal BH Urban CF. Nicotine induces neutrophil extracellular traps. J Leukoc Biol. (2016) 100:1105–12. 10.1189/jlb.3AB0815-379RR 27312847 PMC5069087

[285] Clark HL Abbondante S Minns MS Greenberg EN Sun Y Pearlman E. Protein Deiminase 4 and CR3 regulate aspergillus fumigatus and β-glucan-induced neutrophil extracellular trap formation, but hyphal killing is dependent only on CR3. Front Immunol. (2018) 9:1182. 10.3389/fimmu.2018.01182 29896200 PMC5986955

[286] Gupta S Kaplan MJ. The role of neutrophils and NETosis in autoimmune and renal diseases. Nat Rev Nephrol. (2016) 12:402–13 1. 10.1038/nrneph.2016.71 27241241 PMC5510606

